# Synthesis, enzyme inhibition assay, and molecular modeling study of novel pyrazolines linked to 4-methylsulfonylphenyl scaffold: antitumor activity and cell cycle analysis†

**DOI:** 10.1039/d4ra03902e

**Published:** 2024-07-12

**Authors:** Alaa A.-M. Abdel-Aziz, Adel S. El-Azab, Simone Brogi, Rezk R. Ayyad, Hamad M. Alkahtani, Hatem A. Abuelizz, Ibrahim A. Al-Suwaidan, Abdulrahman M. Al-Obaid

**Affiliations:** a Department of Pharmaceutical Chemistry, College of Pharmacy, King Saud University P. O. Box 2457 Riyadh 11451 Saudi Arabia; b Department of Pharmacy, University of Pisa Via Bonanno 6 56126 Pisa Italy; c Department of Pharmaceutical Chemistry, Faculty of Pharmacy, Al-AzharUniversity Cairo Egypt rezkayyad.222@Azhar.edu.eg

## Abstract

Antitumor activity using 59 cancer cell lines and enzyme inhibitory activity of a newly synthesized pyrazoline-linked 4-methylsulfonylphenyl scaffold (compounds 18a–q) were measured and compared with those of standard drugs. Pyrazolines 18b, 18c, 18f, 18g, 18h, and 18n possessed significant antitumor activity, with a positive cytotoxic effect (PCE) of 22/59, 21/59, 21/59, 48/59, 51/59, and 20/59, respectively. The cancer cell lines HL60, MCF-7, and MDA-MB-231 were used to measure the IC_50_ values of derivatives 18c, 18g, and 18h*via* the MTT assay method, and the results were compared with those of reference drugs. Derivatives 18g and 18h showed potent and broad-spectrum antitumor activities against HL60 (IC_50_ of 10.43, 8.99 μM, respectively), MCF-7 (IC_50_ of 11.7 and 12.4 μM, respectively), and MDA-MB-231 (IC_50_ of 4.07 and 7.18 μM, respectively). Compound 18c exhibited strong antitumor activity against HL60 and MDA-MB-231 cell lines with IC_50_ values of 8.43 and 12.54 μM, respectively, and moderate antitumor activity against MCF-7 cell lines with an IC_50_ value of 16.20 μM. Compounds 18c, 18g, and 18h remarkably inhibited VEGFR2 kinase (IC_50_ = 0.218, 0.168, and 0.135 μM, respectively) compared with the reference drug sorafenib (IC_50_ = 0.041 μM). Compounds 18g and 18h effectively inhibited HER2 kinase (IC_50_ = 0.496 and 0.253 μM, respectively) compared with erlotinib (IC_50_ = 0.085 μM). Compound 18h inhibited EGFR kinase (IC_50_ = 0.574 μM) with a potency comparable with that of the reference drug erlotinib (IC_50_ = 0.105 μM). Pyrazolines 18c, 18f, and 18h arrested the S/G2 phase of the cell cycle in HL-60 cells. In addition, derivatives 18c, 18f, and 18h revealed lower Bcl-2 protein expression anti-apoptotic levels and higher Bax, caspase-3, and caspase-9 expression levels. Molecular docking studies of derivative 18h into the binding sites of EGFR, HER2, and VEGFR2 kinases explored the interaction mode of these pyrazoline derivatives and their structural requirements for antitumor activity.

## Abstract 2

Antitumor activity using 59 cancer cell lines and enzyme inhibitory activity of a newly synthesized pyrazoline-linked 4-methylsulfonylphenyl scaffold (compounds 18a–q) were measured and compared with those of standard drugs.

## Graphical abstract

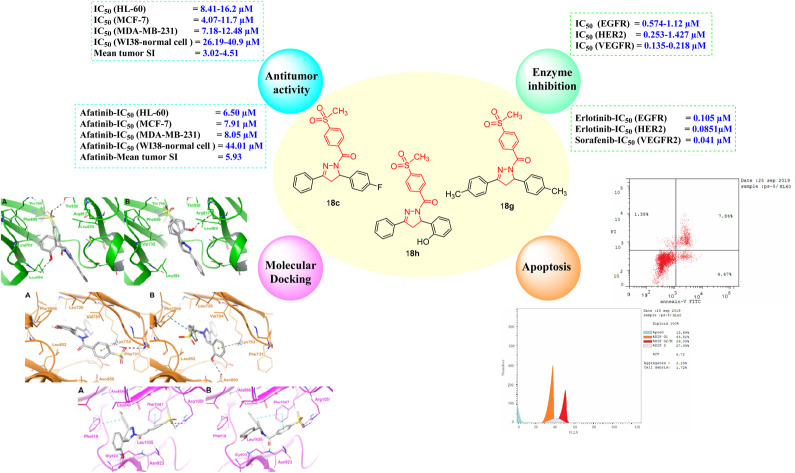

## 1. Introduction

Despite significant advances in cancer patient survival over the last three decades, cancer remains one of the world's most dangerous diseases and one of the leading causes of death.^1–3^ Cancer cells have evolved to become resistant to existing therapies;^4,5^ therefore, there is a great demand for novel and potent anticancer agents.^6–23^ The use of a combination of multiple drugs in cancer therapy has multiple side effects, which can be mitigated using a single compound with multiple molecular mechanisms, which is currently the preferred therapeutic strategy.^24–35^ The vascular endothelial growth factor receptor 2 (VEGFR2), epidermal growth factor receptor (EGFR), and human epidermal growth factor receptor 2 (HER2) are receptor tyrosine kinases that regulate many cell features, including cellular proliferation, cell cycle progression, survival, differentiation, and migration.^36–41^ Overactivity of VEGFR2, EGFR, and HER-2 has been primarily observed in several types of cancer, including breast, colorectal, non-small cellular lung, pancreatic, malignant melanoma, urothelial, B-cell lymphoma, hepatocellular carcinoma, and leukemia.^36–44^

**Fig. 1:**
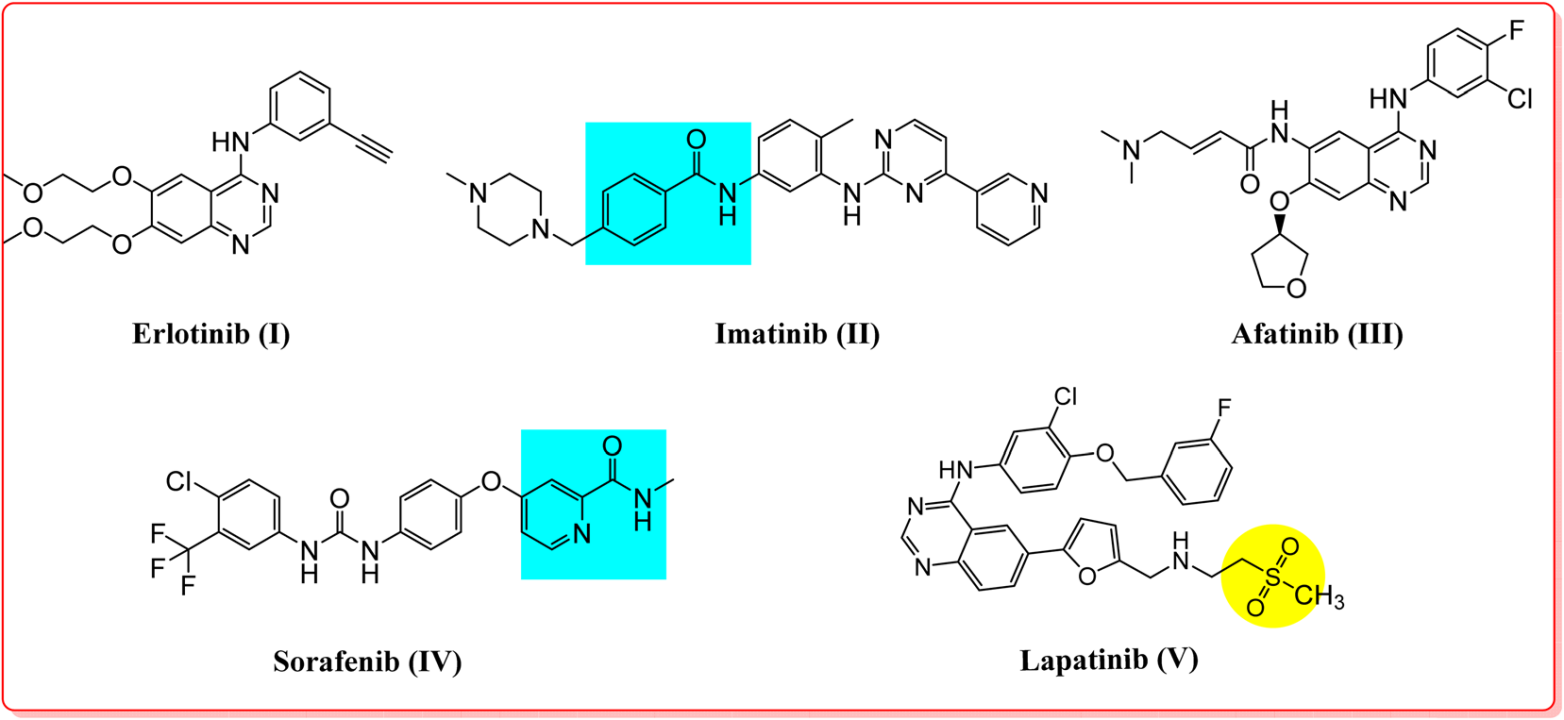
FDA aproved receptor tyrosine kinase inhibitors as anticancer agents.

**Fig. 2:**
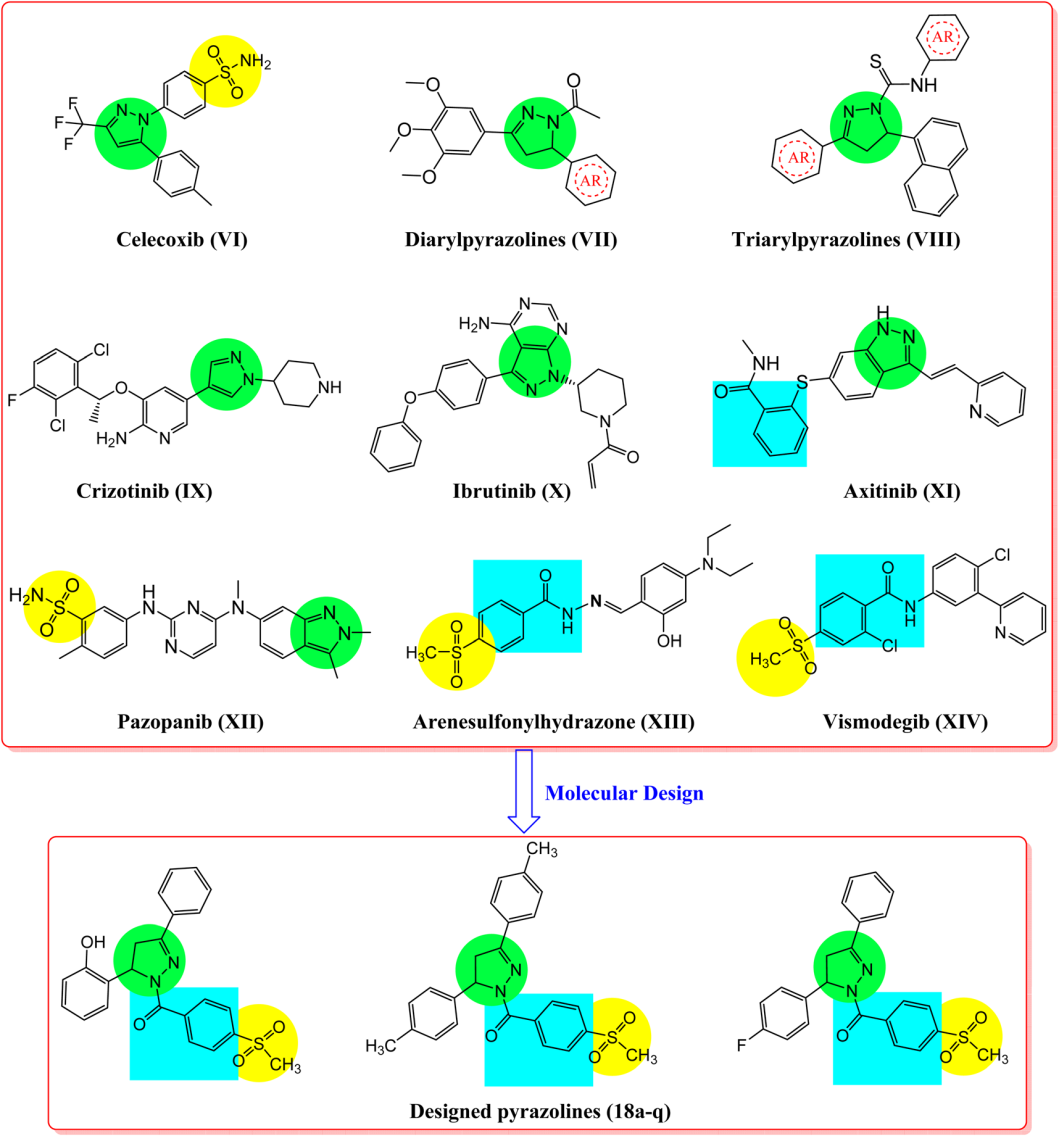
Reported antitumor pyrazole, benzenesulfonyl compounds (VI–XIV), and the designed pyrazole-linked benzenesulfonyl derivatives (18a–q).

In addition, inhibition of these receptor tyrosine kinases initiates apoptosis in leukemia and breast and lung cancers.^36–44^ Various VEGFR2, EGFR, and HER2 inhibitors are potent antitumor agents used to treat cancer.^45–50^ Erlotinib (I), imatinib (II), afatinib (III), sorafenib (IV), and lapatinib (V) are FDA-approved receptor tyrosine kinase inhibitors used for the treatment of various types of cancers (Fig. 1).^45–50^ Moreover, another aspect of some cancer cells is the overexpression of the COX-2 isozyme, and in particular, it was found to be overexpressed in lung, colon, prostate, hepatocellular, ovarian, gastric, and breast cancers, indicating that the COX-2 enzyme could represent a promising drug target for possible antitumor therapy.^51,52^ In some cases, this antitumor effect *via* COX-2 inhibition may proceed through apoptosis.^53^ Accordingly, cancer-associated selective COX-2 inhibitors such as celecoxib (VI) can be used for tumor prevention, and they have also been reported to exhibit activity against prostate tumors in various experimental models of cancer (Fig. 2).^54,55^

On the other hand, compounds incorporating pyrazole and pyrazoline scaffolds possess a variety of biological activities, such as anti-inflammatory, antioxidant, antibacterial, antiviral, and antitumoral activities, including those in lung, liver, colorectal, and breast.^56–65^ Moreover, pyrazoline scaffolds are fundamental for the inhibition of several biological targets, such as carbonic anhydrase, COX-2, EGFR, and HER2, indicating their valuable importance in cancer treatment.^54–61,66–70^ A wide range of compounds incorporating pyrazoles and reduced form pyrazolines have been reported as potent antitumor agents used to prevent cancer.^61–70^ Examples of these compounds are diarylpyrazolines (VII), triarylpyrazolines (VIII), crizotinib (IX), ibrutinib (X), axitinib (XI), and pazopanib (XII), which exhibit excellent anticancer and tyrosine kinase inhibition activities (Fig. 2).^56,71–74^ Meanwhile, compounds containing methylsulfonyphenyl fragments like arenesulfonylhydrazone (XIII) and vismodegib (XIV), have been identified as promising antitumor agents against lung, colon, and liver cancers (Fig. 2).^33,75,76^ The mechanism underlying some of these anticancer agents has been studied using COX-2, EGFR, and HER2 inhibition assays, as well as apoptosis induction testing.^33,36–44,53^

According to the aforementioned rationale, a series of pyrazoline derivatives (compounds 18a–q), incorporating a 4-methylsulfonylbenzene nucleus (Fig. 2), was synthesized. *In vitro* antitumor activities and the structure–activity relationship (SAR) were studied using 59 human cancer cell lines. The inhibitory activity of the most promising compounds against EGFR, HER2, and VEGFR2 kinases, in addition to the COX-2 enzyme, was evaluated. Furthermore, an apoptosis and cell cycle analysis of the most active compounds in the HL-60 cell line was performed. The relationship between Bcl-2 and Bax gene expression and caspase-3 and caspase-9 activation was studied. Molecular docking of target kinase inhibitors was performed to predict their mode of interaction in the binding pockets of EGFR, HER2, and VEGFR2 tyrosine kinases.

## 2. Results and discussion

### 2.1. Chemistry

**Scheme 1:**
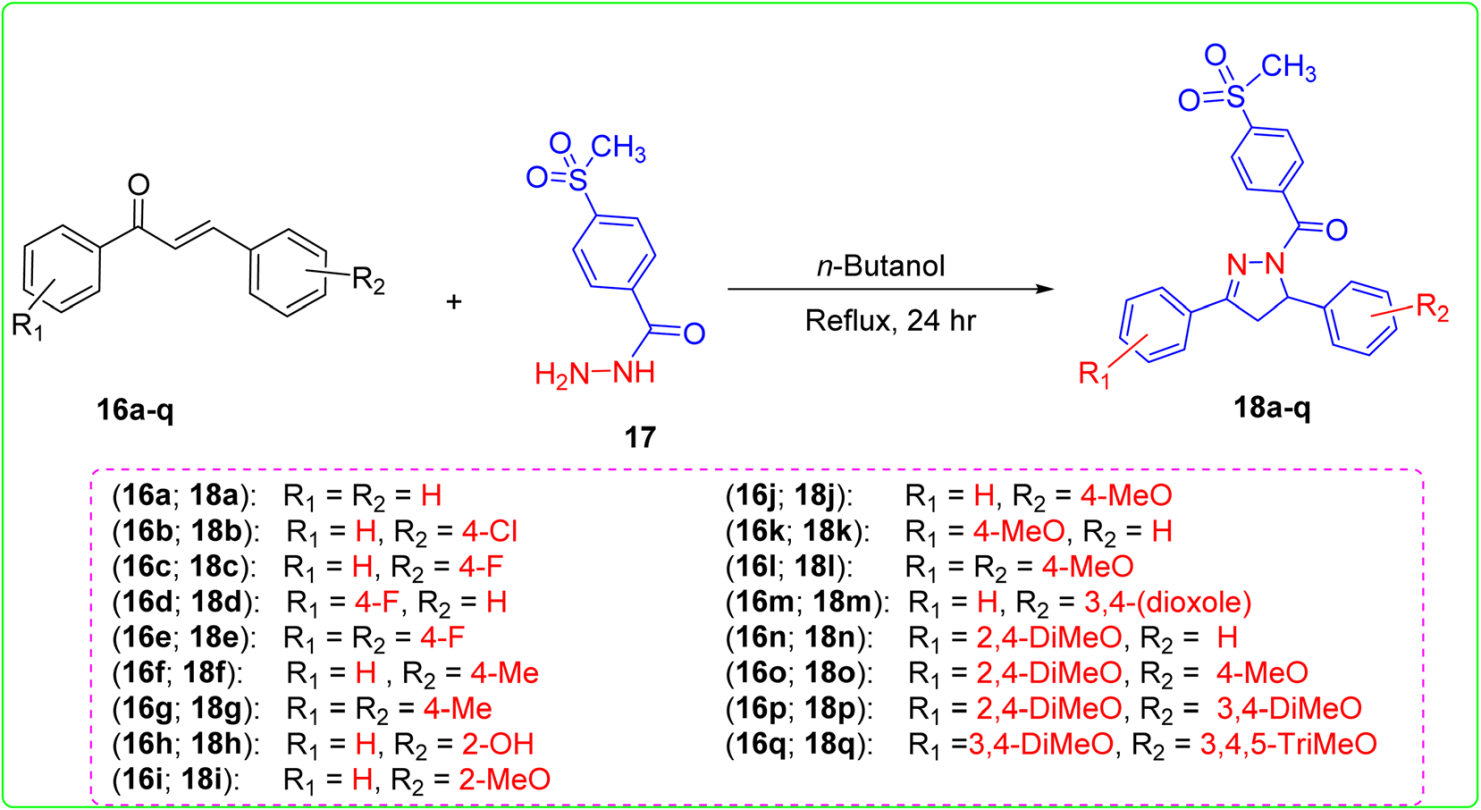
Synthesis of the designed triarylpyrazolines 18a–q.

Chalcones 16a–q and 4-(methylsulfonyl)benzohydrazide (17) reacted in refluxing *n*-butanol for 24 h to produce the pyrazoline derivatives 18a–q, as shown in Scheme 1.^33,58^ The chemical structure of these novel pyrazolines 18a–q was determined by ^1^H and ^13^C nuclear magnetic resonance spectroscopy (NMR), infrared spectroscopy (IR), and mass spectroscopy (MS). FTIR scans of pyrazoline derivatives showed absorption bands (cm^−1^) between 1685 and 1629 (C=O) and 1369 and 1117 (SO_2_). The ^1^H NMR spectra of pyrazolines displayed distinct peaks at chemical shift values ranging from 4.09 to 3.50 ppm and 3.68 to 2.91 ppm. These peaks were identified as a doublet of doublet (dd) and multiplet (m), corresponding to protons at the 4-position. The *J*-coupling constant values for these protons were calculated from 18.6 to 2.4 Hz. The protons located at the 5-position of the pyrazoline rings probably interact with the protons at the 4-position. These interactions are reflected in the signal observed at roughly 5.94–4.09 ppm. The signals exhibit a doublet of doublet (dd), doublet (d), and multiplet (m) patterns, with *J*-coupling constant values ranging from 12.4–2.90 Hz. In ^13^C NMR, two separate signals were found for pyrazoline rings' carbon atoms 4 and 5. These signals had chemical shift values of about 41 and 61 ppm. As in our previous studies, certain synthesized pyrazolines exhibited two distinct s-*cis* and s-*trans* rotamers in their ^1^H NMR and ^13^C NMR spectra.^58^ This can be explained by the slow rotation of the methylsulfonylbenzoyl fragment around the nitrogen atom axis of the pyrazoline-N.^58^ The ^1^H NMR data suggests that the s-*trans* rotamer is more prominent than the s-*cis* rotamer, which can be attributed to the stability of the s-*trans* rotamer, as discussed in our prior work.^58^

### 2.2. Biological evaluation

#### 2.2.1. Antitumor activity

##### 2.2.1.1. Growth inhibition percentage (GI) at a single dose concentration of 10 μM

The National Cancer Institute (NCI), Bethesda, MD, USA, selected compounds 18a–q to evaluate their *in vitro* antitumor activity against a comprehensive collection of 59 cancer cell lines, as shown in Table 1.^33^ Cell lines were obtained from several human tissues, including blood, lung, colon, brain, skin, ovary, kidney, prostate, and breast. The antitumor effectiveness was evaluated using a single dose concentration of 10 μM, and the growth inhibition percentage (GI%) was measured in 59 cancer cell lines. The GI% results were compared with those of imatinib as a reference drug. Compounds 18a–q exhibited significant antitumor activity against the 59 cell lines at a concentration of 10 μM with PCE (ratio between the number of cell lines with percentage growth inhibition ranging from 11 to 100 and the total number of cell lines) of 6/59–51/59 and a percentage mean growth (MG) of 98.11–72.20% (Table 1). These findings were compared with imatinib-GI% as a reference drug. Compounds 18b–h and 18l–o showed the highest PCE of 14/59–51/59 (MG = 95.78–72.20%), whereas compounds 18a, 18i, 18j, 18k, 18p, and 18q showed the lowest PCE of ≤12/59 (MG = 98.11–95.96%) compared with imatinib (PCE = 20/55 and MG = 92.62%). Interestingly, compounds 18b, 18c, 18d, 18f, 18g, 18h, 18m, and 18n were the most active antitumor agents (PCE = 22/59, 21/59, 18/59, 21/59, 48/59, 51/59, 18/59, and 20/59, respectively, and MG = 92.03, 90.58, 93.02, 93.80, 72.98, 72.20, 91.96, and 93.24%, respectively). Compounds 18a, 18e, 18i, 18j, 18k, 18l, 18o, and 18p showed moderate activity (PCE = 11/59, 15/59, 12/59, 10/59, 12/59, 14/59, 15/59, and 11/59, respectively, and MG = 98.11, 94.18, 96.46, 97.16, 97.49, 95.78, 94.17, and 97.33%, respectively).

**Table 1:** Antitumor activity of pyrazolines 18a–q presented as growth inhibition percentages (GI%) using 59 subpanel tumor cell lines

Compound no.	PCE^a^	Cancer cell line assays^b^ (10.0 μM in one dose, GI%)	MG^c^%
18a	11/59	Leukemia (HL-60(TB), 32%; K-562, 19%; MOLT-4, 32%; SR, 13%), non-small cell lung (A549/ATCC, 18%; EKVX, 11%; NCI-H226, 11%; NCI-H522, 18%), melanoma (MALME-3M, 11%; UACC-257, 14%), renal (UO-31, 12%)	98.11
18b	22/59	Leukemia (HL-60(TB), 22%; K-562, 32%; MOLT-4, 17%; RPMI-8226, 11%), NSC lung (A549/ATCC, 13%; EKVX, 21%; HOP-62, 29%; NCI-H226, 15%; NCI-H522, 27%), colon (HCT-116, 26%; HT29, 13%), CNS (SNB-75, 13%), melanoma (UACC-62, 14%), ovarian (SK-OV-3, 26%), renal (CAKI-1, 18%; UO-31, 32%), prostate (PC-3, 22%), breast (MCF7, 13%; MDA-MB-231/ATCC, 11%; BT-549, 14%; T-47D, 31%; MDA-MB-468, 12%)	92.03
18c	21/59	Leukemia (HL-60(TB), 46%; K-562, 33%; MOLT-4, 39%; RPMI-8226, 20%), NSC lung (A549/ATCC, 32%; EKVX, 15%; HOP-62, 26%; NCI-H522, 48%), colon (COLO 205, 22%; HT29, 19%), CNS (SNB-75, 14%; U251, 12%), melanoma (UACC-257, 32%), ovarian (OVCAR-8, 20%; SK-OV-3, 14%), renal (CAKI-1, 22%; UO-31, 20%), prostate (PC-3, 17%), breast (MCF7, 11%; BT-549, 14%; T-47D, 24%)	90.58
18d	18/59	Leukemia (HL-60(TB), 57%; K-562, 32%; MOLT-4, 37%), non-small cell lung (A549/ATCC, 24%; EKVX, 16%; HOP-62, 29%; NCI-H322M, 14%; NCI-H522, 43%), colon (HT29, 14%), CNS (U-251, 17%), melanoma (UACC-257, 37%), ovarian (OVCAR-8, 15%; SK-OV-3, 13%), renal (CAKI-1, 14%; TK-10, 12%; UO-31, 27%), breast (BT-549, 15%; T-47D, 17%)	93.02
18e	15/59	Leukemia (HL-60(TB), 48%; K-562, 29%; MOLT-4, 35%; RPMI-8226, 12%), non-small cell lung (EKVX, 12%; HOP-62, 14%; NCI-H226, 12%; NCI-H522, 15%), colon (HCT-116, 17%), CNS (SNB-75, 18%), renal (CAKI-1, 17%; UO-31, 22%), prostate (PC-3, 15%), (MCF7, 13%; T-47D, 17%)	94.18
18f	21/59	Leukemia (HL-60(TB), 14%; MOLT-4, 30%; RPMI-8226, 18%), NSC lung (A549/ATCC, 16%; EKVX, 13%; HOP-62, 12%; NCI-H226, 17%; NCI-H522, 37%), colon (HCT-116, 20%; HT29, 12%), CNS (SNB-75, 20%; U251, 11%), melanoma (SK-MEL-2, 11%; UACC-257, 26%; UACC-62, 12%), renal (CAKI-1, 16%; UO-31, 24%), prostate (PC-3, 28%), breast (MCF7, 11%; T-47D, 16%; MDA-MB-468, 15%)	93.80
18g	48/59	Leukemia (HL-60(TB), 66%; K-562, 80%; MOLT-4, 56%; SR, 50%), NSC lung (A549/ATCC, 43%; EKVX, 25%; HOP-62, 26%; NCI-H23, 12%; NCI-H322M, 12%; NCI-H460, 17%; NCI-H522, 73%), colon (COLO 205, 33%; HCT-116, 37%; HCT-15, 45%; HT29, 15%; KM12, 37%; SW-620, 25%), CNS (SF-268, 11%; SF-295, 30%; SNB-19, 13%; SNB-75, 30%; U251, 23%), melanoma (LOX IMVI, 16%; MALME-3M, 14%; M14, 32%; MDA-MB-435, 76%; SK-MEL-2, 33%; SK-MEL-28, 23%; SK-MEL-5, 38%; UACC-257, 40%; UACC-62, 36%), ovarian (IGROV1, 23%; OVCAR-3, 24%; OVCAR-4, 22%; NCI/ADR-RES, 38%; SK-OV-3, 26%), renal (786-0, 22%; A498, 16%; ACHN, 12%; CAKI-1, 43%; UO-31, 26%), prostate (PC-3, 33%), breast (MCF7, 52%; MDA-MB-231/ATCC, 14%; HS 578T, 15%; BT-549, 27%; T-47D, 32%; MDA-MB-468, 45%)	72.98
18h	51/59	Leukemia (CCRF-CEM, 41%; HL-60(TB), 73%; K-562, 55%; MOLT-4, 62%; RPMI-8226, 16%; SR, 30%), NSC lung (A549/ATCC, 45%; EKVX, 41%; HOP-62, 47%; NCI-H226, 15%; NCI-H23, 12%; NCI-H322M, 48%; NCI-H460, 54%; NCI-H522, 55%), colon (COLO 205, 20%; HCT-116, 35%; HCT-15, 56%; HT29, 20%; KM12, 21%), CNS (SF-268, 39%; SF-295, 23%; SNB-19, 17%; U251, 48%), melanoma (LOX IMVI, 34%; MALME-3M, 21%; M14, 29%; SK-MEL-2, 28%; SK-MEL-28, 11%; SK-MEL-5, 38%; UACC-257, 39%; UACC-62, 12%), ovarian (IGROV1, 28%; OVCAR-3, 35%; OVCAR-4, 22%; OVCAR-8, 41%; NCI/ADR-RES, 22%; SK-OV-3, 25%), renal (786-0, 33%; ACHN, 25%; CAKI-1, 33%; RXF 393, 29%; SN12C, 17%; TK-10, 25%; UO-31, 47%), prostate (PC-3, 21%; DU-145, 19%), breast (MCF7, 22%; MDA-MB-231/ATCC, 32%; BT-549, 19%; T-47D, 30%; MDA-MB-468, 17%)	72.20
18i	12/59	Leukemia (HL-60(TB), 46%; MOLT-4, 28%), non-small cell lung (A549/ATCC, 35%; EKVX, 12%; HOP-62, 20%; NCI-H226, 13%; NCI-H522, 29%), colon (HT-29, 17%), melanoma (UACC-257, 36%), renal (CAKI-1, 23%; UO-31, 28%), breast (T-47D, 17%)	96.46
18j	10/59	Leukemia (HL-60(TB), 35%; K-562, 13%; MOLT-4, 26%), non-small cell lung (A549/ATCC, 24%; EKVX, 12%; NCI-H226, 11%; NCI-H522, 14%), CNS (SNB-75, 12%), melanoma (UACC-257, 14%), breast (T-47D, 16%)	97.16
18k	12/59	Leukemia (HL-60(TB), 18%; MOLT-4, 27%; RPMI-8226, 11%), non-small cell lung (A549/ATCC, 13%; NCI-H226, 14%; NCI-H522, 35%), colon (HT-29, 20%), CNS (SNB-75, 11%), melanoma (K-MEL-5, 14%), renal (UO-31, 15%), prostate (PC-3, 21%), breast (MDA-MB-468, 16%)	97.49
18l	14/59	Leukemia (HL-60(TB), 47%; K-562, 18%; MOLT-4, 33%), non-small cell lung (A549/ATCC, 40%; HOP-62, 11%; NCI-H522, 34%), colon (HT-29, 19%), CNS (SNB-75, 11%), melanoma (UACC-257, 45%), renal (CAKI-1, 14%; UO-31, 16%), breast (MCF7, 11%; BT-549, 13%; T-47D, 11%)	95.78
18m	18/59	Leukemia (HL-60(TB), 63%; K-562, 40%; MOLT-4, 48%), NSC lung (A549/ATCC, 20%; HOP-62, 17%; NCI-H226, 11%; NCI-H522, 13%), colon (COLO 205, 19%; HCT-116, 18%; HCT-15, 14%), CNS (SNB-75, 12%), ovarian (SK-OV-3, 16%), renal (A498, 14%; UO-31, 34%), prostate (PC-3, 15%), breast (MCF7, 16%; BT-549, 14%; T-47D, 22%)	91.96
18n	20/59	Leukemia (HL-60(TB), 55%; K-562, 26%; MOLT-4, 38%), NSC lung (EKVX, 28%; HOP-62, 20%; NCI-H226, 14%; NCI-H522, 20%), colon (HCT-116, 18%; KM12, 12%), CNS (SNB-75, 15%), melanoma (SK-MEL-2, 18%; SK-MEL-5, 21%), ovarian (OVCAR-4, 12%), renal (CAKI-1, 15%; UO-31, 24%), prostate (PC-3, 15%), breast (MCF7, 65%; MDA-MB-231/ATCC, 12%; T-47D, 14%; MDA-MB-468, 36%)	93.24
18o	15/59	Leukemia (HL-60(TB), 51%; K-562, 32%; MOLT-4, 40%; SR, 11%), NSC lung (A549/ATCC, 31%; HOP-62, 18%; NCI-H522, 43%), colon (HT29, 17%), CNS (SNB-75, 15%), melanoma (SK-MEL-5, 12%; UACC-257, 38%), renal (CAKI-1, 12%; UO-31, 19%), breast (BT-549, 11%; T-47D, 18%)	94.17
18p	11/59	Leukemia (HL-60(TB), 31%; K-562, 23%), NSC lung (HOP-62, 26%; NCI-H226, 15%; NCI-H522, 13%), colon (HCT-116, 17%), CNS (SF-268, 11%), ovarian (SK-OV-3, 16%), renal (UO-31, 25%), breast (T-47D, 25%; MDA-MB-468, 13%)	97.33
18q	6/59	CNS (SNB-75, 13%), renal (A498, 13%; CAKI-1, 13%; UO-31, 24%), breast (MCF7, 12%; MDA-MB-468, 12%)	97.51
Imatinib	20/59	Leukemia (MOLT-4, 18%; PRMI-8226, 12.6%; SR, 14.6%), NSC lung (EKVX, 15.7%; NCI-H226, 10.6%; NCI-H23, 17.1%), colon (HCT-116, 18.6%; HCT-15, 11.5%; HT-29, 47.1%), CNS (SF-295, 15.1%; SF-539, 24.5%; U251, 10.6%), melanoma (LOX IMVI, 11.6%; SK-MEL-5, 22.3%), renal (A-498, 13.7%), prostate (PC-3, 10.6%; DU-145, 14.4%), breast (MDA-MB-231/ATCC, 11.2%; T-47D, 18.6%; MDA-MB-468, 29.1%)	92.62%

a PCE: positive cytotoxic effect; the ratio between the number of cell lines with percentage growth inhibition >10% and the total cell lines.

b MGI%: mean growth inhibition percent.

c MG%: mean growth percent.

The broad-spectrum and selectivity of compounds 18a–q (Table 1) against the 59 cell lines showed that compounds 18b, 18c, 18d, 18e, 18f, 18g, 18h, 18m, 18n, and 18o had significant GI (>10–80%) against most of the cancer cell lines tested [leukemia, non-small cell lung cancer (NSCLC), melanoma, colon, CNS, ovarian, renal, prostate, and breast cancer] compared with imatinib (GI% < 10–47). Compounds 18b, 18c, 18d, 18f, 18g, 18h, 18m, and 18n showed significant antitumor activity against leukemia (GI% = 11–80), NSCLC (GI% = 11–73), colon cancer (GI% = 12–56), CNS cancer (GI% = 11–48), melanoma (GI% = 11–76), ovarian cancer (GI% = 12–41), renal cancer (GI% = 12–47), prostate cancer (GI% = 15–33), and breast cancer (GI% = 11–65). In contrast, the antitumor activity of imatinib was moderate against leukemia (GI% = 13–18), NSCLC (GI% = 11–17), colon cancer (GI% = 12–47), CNS cancer (GI% = 11–25), melanoma (GI% = 12–22), ovarian cancer (GI% < 10), renal cancer (GI% < 10–14), prostate cancer (GI% = 11–14), and breast cancer (GI% = 11–29).

##### 2.2.1.2. Structure–activity relationship study (Table 1)

Structure correlation analysis showed that the 2-hydroxyphenyl derivative 18h (PCE = 51/59) had significant and potent antitumor activity compared with unsubstituted phenyl and 2-methoxyphenyl derivatives, such as compounds 18a and 18i (PCE = 11/59 and 12/59, respectively). Replacement of the phenyl moiety of compound 18a with a 4-tolyl fragment, such as in compound 18f, increased antitumor activity (PCE = 11/59 and 21/59, respectively). In contrast, replacement of the 4-tolyl compound 18f (PCE = 21/59) with the corresponding halogenated derivatives, such as compounds 18b, 18c, and 18d, retained antitumor activity (PCE = 22/59, 21/59, and 18/59, respectively). The introduction of two tolyl groups, such as compound 18g, led to a sharp increase in antitumor activity (PCE = 48/59) compared with unsubstituted phenyl and 4-tolyl derivatives, such as compounds 18a and 18f (PCE = 11/59 and 21/59, respectively). The 4-tolyl and bis-4-tolyl compounds 18f and 18g showed significant antitumor activity (PCE = 21/59 and 48/59, respectively) compared with the corresponding 4-methoxyphenyl derivatives (compounds 18j and 18k) and bis-4-methoxyphenyl (compound 18l) (PCE = 10/59, 12/59, and 14/59 respectively). Derivatives incorporating the 2,4-dimethoxy phenyl moiety, such as compound 18n (PCE = 20/59), were potent antitumor agents compared with the corresponding 2-methoxy and 4-methoxy compounds 18i, 18j, 18k, and 18l (PCE = 12/59, 10/59, 12/59, and 14/59, respectively). Introduction of more than three methoxy groups at the phenyl fragment, such as in compounds 18p and 18q, did not improve antitumor activity (PCE = 11/59 and 6/59, respectively) compared with the unsubstituted phenyl compound 18a (PCE = 11/59). Replacement of the 4-methoxyphenyl moiety, such as compound 18j, with a 3,4-piperonyl derivative, such as compound 18m, increased antitumor activity (PCE = 10/59 and 18/59, respectively). Insertion of a halogen atom into compound 18a (PCE = 11/59) significantly increased the antitumor activity of compounds 18b, 18c, and 18d (PCE = 22/59, 21/59, and 18/59, respectively).

##### 2.2.1.3. IC_50_ of some selected compounds 18c, 18g, and 18h against human leukemia and breast cancer cells

The IC_50_ value of selected antitumor compounds 18c, 18g, and 18h were measured using leukemia HL60, breast adenocarcinoma MCF-7, and MDA-MB-231 cells at various concentrations *via* the standard MTT assay method using afatinib, staurosporine, and doxorubicin (DOX) as reference drugs (Table 2).^77^ Compound 18g showed strong antitumor activity against HL-60 (IC_50_ = 10.43 μM), MCF-7 (IC_50_ = 11.70 μM), and MDA-MB-231 (IC_50_ = 4.07 μM). Similarly, compound 18h exerted broad-spectrum and strong antitumor activity against the three cancer cell lines HL-60, MCF-7, and MDA-MB-231 with IC_50_ values of 8.99, 12.48, and 7.18 μM, respectively. Compound 18c exhibited strong antitumor activity against the cancer cell lines HL-60 (IC_50_ = 8.43 μM) and MDA-MB-231 (IC_50_ = 12.54 μM), and moderate activity against MCF-7 cells (IC_50_ = 16.2 μM).

**Table 2:** *In vitro* cytotoxic study (IC_50_ values μM) of the target compounds 18c, 18g, and 18h against three cancer cell lines and WI38 normal cell line

Compd no.	Mean GI%		*In vitro* cytotoxicity^a^ IC_50_ (μM)				Mean tumor selectivity index^b^
	Leukemia	Breast cancer	HL-60	MCF-7	MDA-MB-231	WI38	
18c	35	16	8.43 ± 1.7	16.2 ± 0.72	12.54 ± 0.56	34.81 ± 1.54	3.02
18g	63	31	10.43 ± 0.68	11.7 ± 0.96	4.07 ± 0.18	26.19 ± 1.16	3.73
18h	46	24	8.99 ± 0.4	12.48 ± 0.55	7.18 ± 0.32	40.9 ± 1.81	4.51
Afatinib	—	—	6.50 ± 0.77	7.91 ± 0.66	8.05 ± 0.72	44.01 ± 1.9	5.93
Staurosporine	—	—	5.191 ± 0.23	3.841 ± 0.17	5.814 ± 0.26	20.23 ± 0.9	4.21
DOX	—	—	6.01 ± 0.2	4.77 ± 0.4	5.10 ± 0.31	7.12 ± 0.7	1.36

a IC_50_ value is the concentration of compound that inhibits 50% of the cancer cell growth after 48 h of drug exposure, as obtained from the MTT assay. Each value was shown as mean ± SD of three experiments.

b Mean GI% and tumor selectivity index (WI38/HL60, MCF7, and MDA-MB-231).

##### 2.2.1.4. *In vitro* cytotoxicity against normal human cell

A normal fibroblast-like fetal lung cell line (WI-38) was used to further investigate the therapeutic safety of the newly synthesized hybrids and evaluate their selective cytotoxicity toward normal and tumor cells.^33,77^ For comparison, afatinib, staurosporine, and DOX were used as standard anticancer drugs. As shown in Table 2, the investigated compounds exhibited lower cytotoxicity against WI-38 normal fibroblast cells, as denoted by their IC_50_ values with a range of 26.19–40.9 μM and a mean tumor selectivity index of 3.02–4.51, compared with afatinib, staurosporine, and DOX on normal cells IC_50_ values with a range of 7.12–44.01 μM and a mean tumor selectivity index of 1.36–5.93. Notably, derivatives 18c, 18g, and 18h induced lower toxic effects on WI-38 cells, with IC_50_ values of 34.81, 26.19, and 40.9 μM, respectively, compared with those induced by afatinib (IC_50_ = 44.01 μM), staurosporine (IC_50_ = 20.23 μM), and DOX (IC_50_ = 7.12 μM).

#### 2.2.2. EGFR, HER, VEGFR2, and COX-2 inhibition activities

The inhibitory effects of the selected active compounds 18c, 18g, and 18h against EGFR, HER2, VEGFR2, and COX-2 were investigated and compared with those of the reference drugs erlotinib, sorafenib, and celecoxib (Table 3).^33,60,77^ The IC_50_ values of erlotinib against EGFR and HER2 were 0.105 and 0.085 μM, respectively, whereas the IC_50_ of sorafenib against VEGFR2 was 0.041 μM. The IC_50_ value of celecoxib against the COX-2 enzyme was 2.80 μM. The inhibitory activity of derivatives 18c, 18g, and 18h against EGFR, HER2, and VEGFR2 kinases was in the range of 0.135–1.427 μM.

**Table 3:** *In vitro* inhibitory effects of the pyrazolines 18c, 18g, and 18h against COX-2, EGFR, HER2, and VEGFR2^a^

Compound no.	IC_50_^a^ (μM)			
	COX-2 inhibition	EGFR inhibition	HER2 inhibition	VEGFR2 inhibition
18c	126.54 ± 8.23	1.120 ± 0.037	1.427 ± 0.047	0.218 ± 0.005
18g	94.01 ± 4.41	1.015 ± 0.034	0.496 ± 0.016	0.168 ± 0.003
18h	19.32 ± 0.72	0.574 ± 0.019	0.253 ± 0.008	0.135 ± 0.009
Celecoxib	2.80 ± 0.079	—	—	—
Erlotinib	—	0.105 ± 0.035	0.085 ± 0.003	—
Sorafenib	—	—	—	0.041 ± 0.002

a IC_50_ value is the compound concentration required to produce 50% inhibition. Each value was shown as mean ± SD of three experiments.

Additionally, these compounds exhibited inhibitory effects against COX-2 with IC_50_ in the 19.32–126.54 μM range. Compounds 18g and 18h showed the highest and most potent inhibitory activity against HER2 (IC_50_ = 0.496 and 0.253 μM, respectively) and VEGFR2 (IC_50_ = 0.168 and 0.135 μM, respectively) compared with erlotinib (HER2-IC_50_ = 0.085 μM) and sorafenib (VEGFR2-IC_50_ = 0.041 μM). The derivative 18h showed the highest EGFR inhibitory effect against EGFR (IC_50_ = 0.574 μM) compared to erlotinib (IC_50_ = 0.105 μM). Compound 18c was the least active against EGFR and HER2 (IC_50_ = 1.12 and 1.427 μM, respectively), whereas it was more effective against VEGFR2 (IC_50_ = 0.218 μM). On the other hand, compounds 18c, 18g, and 18h showed ineffective inhibitory activity against the COX-2 enzyme (IC_50_ = 126.54, 94.01, and 19.32 μM, respectively) compared with celecoxib (IC_50_ = 2.80 μM). It is clear that the compound with a 4-tolyl moiety fragment (compound 18g) and the derivative with a 2-hydroxyphenyl moiety (compound 18h) are more potent than the corresponding compound with a 4-fluorophenyl moiety (compound 18c).

#### 2.2.3. Annexin V-FITC apoptosis assay

**Fig. 3:**
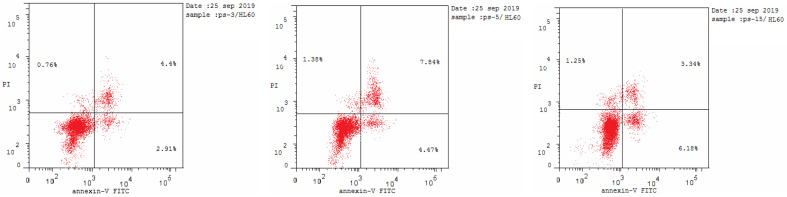
Effect of pyrazolines 18c (left panel), 18g (middle panel), and 18h (right panel) on the percentage of annexin V-FITC-positive staining in HL-60 cells.

Most anticancer agents kill cancer cells *via* an apoptosis induction mechanism.^33,77^ Cytometry was performed to differentiate the apoptosis and necrosis modes of HL-60 cell death induced by the selected active compounds 18c, 18g, and 18h using an annexin V-fluorescein isothiocyanate (FITC)/propidium iodide (AV/PI) dual-staining assay with the BD FACSCalibur (BD Biosciences, San Jose, CA, USA). The results of treating derivatives 18c, 18g, and 18h with HL-60 cells for 24 h at a concentration equal to their IC_50_ values are shown in Table 4 and Fig. 3. An increase in early apoptosis was observed from 0.57% in the control sample (DMSO) to 2.91–6.18% and a sharp increase in late apoptosis from 0.22% to 3.34–7.84%. These data support the apoptotic mechanism underlying programmed cell death induced by compounds 18c, 18g, and 18h rather than the necrotic pathway.

**Table 4:** The effect of compounds 18c, 18g, and 18h and DMSO on the percentage of HL-60 cells stained positive for annexin V-FITC at their IC_50_ concentrations

Compound no.	Apoptosis			Necrosis
	Total	Early	Late	
18c	8.06	2.91	4.4	0.76
18g	13.69	4.47	7.84	1.38
18h	10.77	6.18	3.34	1.25
DMSO^a^	1.71	0.57	0.22	0.92

a Ref. 33.

#### 2.2.4. *In vitro* cell cycle analysis

**Fig. 4:**
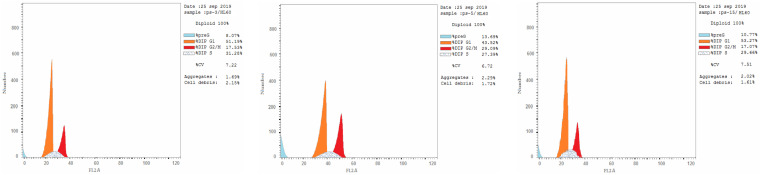
Cell cycle analysis of HL-60 cells treated with pyrazolines 18c (left panel), 18g (middle panel), and 18h (right panel).

Flow cytometry measures cell growth in different cell cycle phases (G0–G1, S, G2/M and pre-G1).^33,77^ This study aimed to find out how the target compounds affect the growth of cancer cells by examining at how they change the cell cycle and cause apoptosis in HL-60 cells. The selected active compounds 18c, 18g, and 18h were used to analyze their effects on cell cycle progression in the HL-60 cell line (Table 5 and Fig. 4), and DMSO was used as a negative control. The HL-60 cells were treated with 18c, 18g, and 18h at concentrations equal to their IC_50_ values to suppress cell growth. The results exhibited a significant effect on the percentage of apoptotic cells, as indicated by an increase in cells in the pre-G1 phase (8.07–13.69%) compared with 1.71% in untreated cells. The percentage of cells in the S phase (27.39–31.28%) and G2/M phase (17.07–29.09%) significantly increased compared with the control (1.71–12.03%), causing cell cycle arrest. In contrast, the percentage of cells in the G0/G1 phase decreased (43.52–53.27%) compared with the control (52.67%). These results indicate that derivatives 18c, 18g, and 18h stop the cell cycle of HL-60 cells at the S/G2 phase, which suggests that they may help prevent the growth of cancer cells.

**Table 5:** Effect of compounds 18c, 18g, and 18h and DMSO on the cell cycle of HL-60 cells at thier IC_50_ concentartions

Compound no.	% G0–G1	% S	% G2–M	% Pre-G1
18c	51.19	31.28	17.53	8.07
18g	43.52	27.39	29.09	13.69
18h	53.27	29.66	17.07	10.77
DMSO^a^	52.67	1.71	12.03	1.71

a Ref. 33.

#### 2.2.5. Apoptotic regulators (Bax, Bcl-2, caspase-3, and caspase-9)

In this study, MDA-MB-231 cells were used along with derivatives 18c, 18g, and 18h at their IC_50_ concentrations. The levels of Bax (apoptosis promoter), Bcl-2 (apoptosis inhibitor), and apoptosis coordination enzymes (caspase-3 and caspase-9) were measured.^78–80^ As shown in Table 6, MDA-MB-231 cells treated with compounds 18c, 18g, and 18h possessed higher levels of Bax protein expression than untreated control cells by fold changes of 4.413, 3.054, and 2.154, respectively, compared with those treated with staurosporine with a fold change of 6.104. In contrast, treatment of compounds 18c, 18g, and 18h with MDA-MB-231 remarkably reduced expression of the apoptosis inhibitor (Bcl-2 protein) with fold changes of 0.184, 0.252, and 0.423, respectively, compared with that of staurosporine with a fold change of 0.249. Moreover, levels of caspase-3 and caspase-9 were effectively increased after treatment of MDA-MB-231 cells with derivatives 18c (fold changes of 7.453 and 5.158, respectively) 18g, (fold changes of 3.945 and 4.386, respectively), and 18h (fold changes of 5.559 and 2.799, respectively) compared with the untreated control. In addition, the levels of caspase-3 and caspase-9 were increased when using the reference drug staurosporine, with fold changes of 7.594 and 5.456, respectively.

**Table 6:** Effect of compounds 18c, 18g, and 18h and DMSO on Bax, Bcl-2, and caspases-3/-9 levels inside MDA-MB-231 cells treated with the compounds at their IC_50_ concentrations

Gene expression (fold change)				
Compound no.	Casp3	Casp9	Bax	Bcl2
18c	7.453	5.158	4.413	0.184
18g	3.945	4.386	3.054	0.252
18h	5.559	2.799	2.154	0.423
Staurosporine	7.594	5.456	6.104	0.249
Control	1	1	1	1

### 2.3. Computational studies

**Fig. 5:**
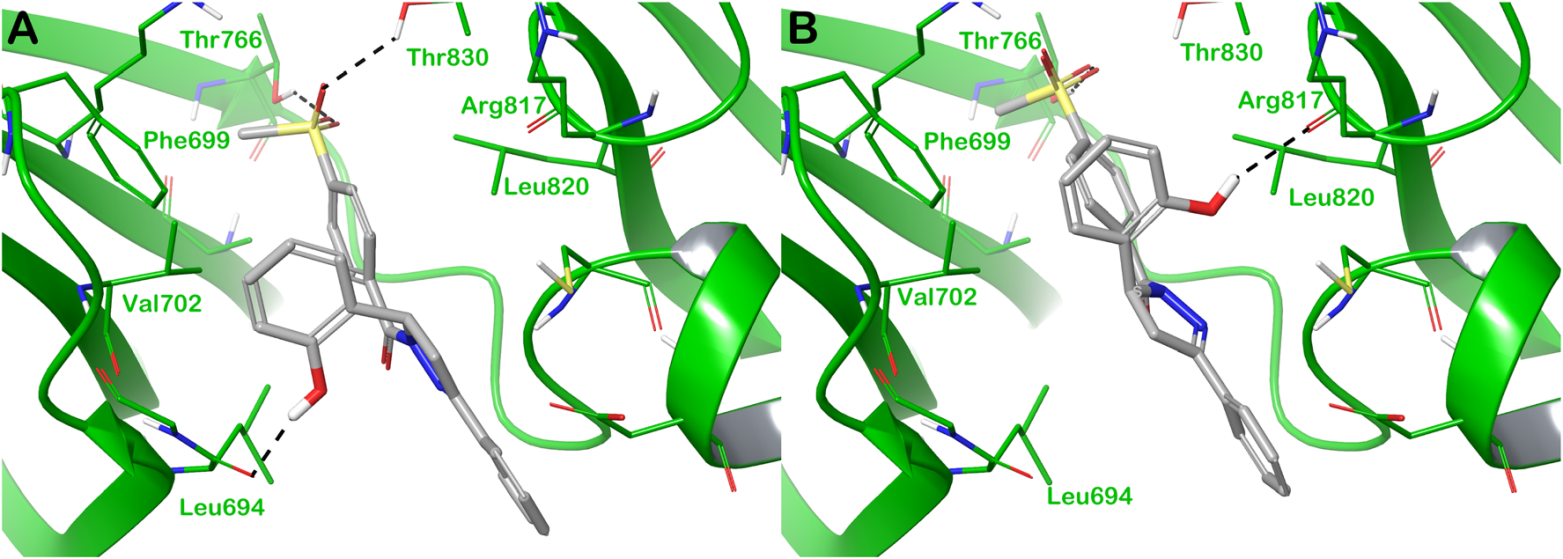
Binding mode of compound 18h (grey sticks) within hEGFR (green cartoon PDB ID 1M17). In panel (A), the R-enantiomer is reported, and in panel (B), the S-enantiomer of the compound is reported. Residues in the binding sites are represented by thin sticks, and hydrogen bonds are shown as grey dotted lines. Images were generated using Maestro (Schrödinger, LLC, New York, 2020).

To investigate the potential binding mode of compound 18h within the selected targets, for which a significant inhibitory capacity has been demonstrated using *in vitro* tests, we conducted an *in silico* analysis mainly based on molecular docking calculations. In particular, considering that the compound was tested as a racemic mixture, our focus was to assess if there were stereoselective interactions within the selected targets in terms of scores and number of contacts. Starting from the protein hEGFR, for which the docking output is shown in Fig. 5, we reported the observation arose from computational studies. In particular, the R-enantiomer of compound 18h (R-18h), within the binding site of hEGFR, established significant contacts, mainly polar contacts (Fig. 5A).

This enantiomer established H-bonds with the backbone of Leu694 and the side chains of Thr766 and Thr830. In addition, hydrophobic contacts (van der Waals interactions) were observed with residues Phe699, Val702, and Leu820. This binding mode accounted for a docking score of −7.983 kcal mol^−1^. The S-enantiomer of compound 18h (S-18h) maintained the hydrophobic interactions found for compound R-18h, whereas the H-bond with the backbone of the reside Leu694 was not detected. However, the hydroxyl group was able to target the backbone of Arg817 by an H-bond, and another H-bond was observed with the side chain of Thr766 (Fig. 5B). The contact with the residues Thr830 found for R-18h was completely lost considering the molecule S-18h. This binding mode with only one interaction of difference in terms of the number of contacts compared with R-18h accounted for a docking score slightly different with respect to that found for R-18h (−7.772 kcal mol^−1^), hypothesizing that there is no present a significant stereoselectivity in terms of interaction with the selected target and the relevant inhibitory activity could be attributed to both enantiomers.

**Fig. 6:**
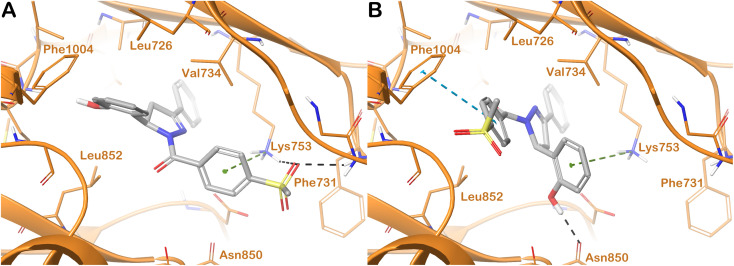
Binding mode of compound 18h (grey sticks) within hHER2 (orange cartoon PDB ID 3RCD). In panel (A), the R-enantiomer is reported, and in panel (B), the S-enantiomer of the compound is reported. Residues in the binding sites are represented by thin sticks, hydrogen bonds are shown as black dotted lines, and the cation–π interaction and the π–π interaction as green and cyan dotted lines, respectively. Images were generated using Maestro (Schrödinger, LLC, New York, 2020).

The docking output for both enantiomers of compound 18h, considering the drug target hHER2, is shown in Fig. 6. R-18h (Fig. 6A) established two H-bonds with the side chain of Lys753 and the backbone of Phe731. This latter residue was also targeted by a cation–π stacking.

Additional hydrophobic contacts with residues Leu726, Val734, Leu852, and Phe1004 were also detected. The mentioned hydrophobic interactions and the cation–π stacking with Lys753 were also found when we considered S-18h (Fig. 6B). For this enantiomer, we observed the lack of two contacts found for R-18h and the replacement of those contacts with two other different interactions, *i.e.*, Asn830 (H-bond) and Phe1004 (π–π stacking). There was no difference between the two enantiomers in terms of the number of contacts established within the selected binding site, and we observed only a slight variation in the docking scores (R-18h = −7.539 kcal mol^−1^; S-18h = −7.715 kcal mol^−1^), indicating a similar contribution to the activity of the racemic mixture from the two enantiomers.

**Fig. 7:**
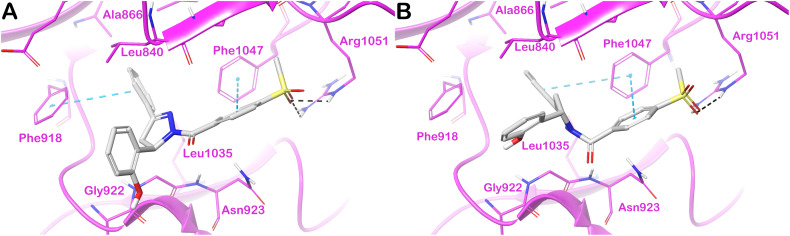
Binding mode of compound 18h (grey sticks) within hVEGFR2 (magenta cartoon PDB ID 4ASD). In panel (A), the R-enantiomer is reported, and in panel (B), the S-enantiomer of the compound is reported. Residues in the binding sites are represented by thin sticks, hydrogen bonds are shown as black dotted lines, and the π–π interaction as cyan dotted lines. Images were generated using Maestro (Schrödinger, LLC, New York, 2020).

Finally, the docking output for the drug target hVEGFR2 is shown in Fig. 7. Panel A illustrates the docking output for R-18h. The considered molecule established two π–π interactions with Phe918 and Phe1047 and H-bonds with the residue Arg1051. Additional hydrophobic contacts with Leu840, Gly922, and Leu1035 were detected. Considering S-18h, we found the same number of contacts described for compound R-18h with the same targeted residues, although the π–π interaction with Phe918, found for R-18h, was replaced by an additional π–π stacking with Phe1047. Due to the similar targeted residues and binding modes between R-18h and S-18h, we found very close docking scores (R-18h = −7.651 kcal mol^−1^; S-18h = −7.608 kcal mol^−1^), probably reflecting the lack of stereoselective interaction of 18h with the selected target. In summary, we can speculate about the importance of the contribution of both enantiomers in the significant inhibitory capacity of compound 18h against the selected proteins.

## 3. Conclusion

A series of pyrazoline derivatives incorporating a methylsulfonylphenyl fragment was synthesized and their antitumor activity was evaluated using a single dose of 10 μM in a full NCI 59-cell line panel assay 18a–q. Pyrazoline derivatives possessing halogenated phenyl fragments (18b–c), tolyl fragments (18f and 18g), and a 2-hydroxyphenyl fragment (18h) exhibited the highest cytotoxic effects among the tested compounds (PCE = 21/59–51/59). Compounds 18g and 18h showed broad-spectrum antitumor activity against HL-60 (IC_50_ of 10.43 and 8.99 μM, respectively), MCF-7 (IC_50_ of 11.70 and 12.48 μM, respectively), and MDA-MB-231 (IC_50_ of 4.07 and 7.18 μM, respectively). Compound 18c showed remarkable antitumor activity in HL-60 and MDA-MB-231 cell lines (IC_50_ = 8.43 and 12.54 μM, respectively) and moderate activity with MCF-7 (IC_50_ = 16.2 μM). The derivatives 18c, 18g, and 18h effectively inhibited VEGFR2 kinase with IC_50_ values of 0.218, 0.168, and 0.135 μM, respectively, compared with the reference drug sorafenib (IC_50_ = 0.041 μM). Compounds 18g and 18h were effective HER2 inhibitors (IC_50_ = 0.496 and 0.0.253 μM, respectively), compared with erlotinib (IC_50_ = 0.085 μM). Compound 18h showed notable inhibition of EGFR kinase (IC_50_ = 0.574 μM) relative to the erlotinib reference drug (IC_50_ = 0.105 μM). The pyrazolines 18c, 18f, and 18h stopped cell growth at the S/G2 phase through pre-G1 apoptosis in the HL-60 cells and induced an increase at the early and late apoptosis stages with total apoptosis percentages of 8.06–13.69 greater than the untreated cells (1.71). Molecular docking studies of the most active pyrazoline 18h indicated the potential of this class of compounds as kinase inhibitor candidates.

## 4. Experimental

### 4.1. Chemistry

The melting points (uncorrected) were measured using a Barnstead 9100 electrothermal melting apparatus from APS Water Services Corporation, located in Van Nuys, CA, USA. The IR spectra were acquired using a PerkinElmer FT-IR spectrometer produced by PerkinElmer Inc., situated in Waltham, MA, USA. The NMR study was performed in DMSO-d6 using Bruker 500 and 700 MHz instruments (Bruker, Billerica, MA, USA), with TMS used as the internal standard. Chemical shifts were reported in *δ* ppm. The mass spectra were obtained utilising an Agilent 6320 Ion Trap mass spectrometer produced by Agilent Technologies in Santa Clara, CA, USA. An analysis of carbon (C), hydrogen (H), and nitrogen (N) was performed at the Research Centre of the College of Pharmacy, King Saud University, Saudi Arabia. The obtained results were found to be within a range of ±0.4% deviation from the theoretical values. Chalcones 1–16 were synthesized using the Claisen–Schmidt condensation method as described in previous studies.^33,58^

#### 4.1.1. General procedure for the synthesis of triarylpyrazolines 18a–q (Scheme 1)

The chalcones 1–16 (10 mmol) were dissolved in 10 mL of *n*-butanol. Then, 4-(methylsulfonyl)benzohydrazide (17) (10 mmol) was added to the solution. The resulting mixture was heated under reflux for 24 h. The progress of the reaction was monitored using thin-layer chromatography (TLC). Once the reaction was complete, the mixture was cooled. The precipitate obtained at a low temperature was subjected to filtration, drying, and recrystallisation using a suitable solvent to obtain pyrazoline derivatives 18a–q.

##### 4.1.1.1. (3,5-Diphenyl-4,5-dihydro-1*H*-pyrazol-1-yl)(4-(methylsulfonyl)phenyl)methanone (18a)

M.P. 259–261 °C, 81% yield (CH_3_OH); IR (KBr, cm^−1^) *ν*: 1637 (C=O), 1294, 1143 (O=S=O); ^1^*H* NMR (500 MHz, CDCl_3_) *δ* 8.19 (d, *J* = 7.9 Hz, 2*H*), 8.05 (d, *J* = 8.1 Hz, 2*H*), 7.72 (d, *J* = 7.1 Hz, 2*H*), 7.50–7.43 (m, 3*H*), 7.41–7.29 (m, 5*H*), 5.85 (dd, *J* = 11.7, 4.8 Hz, 1*H*), 3.87 (dd, *J* = 17.7, 11.8 Hz, 1*H*), 3.30 (dd, *J* = 17.7, 4.8 Hz, 1*H*), 3.12 (s, 3*H*). ^13^C NMR (126 MHz, CDCl_3_) *δ* 164.65, 155.95, 142.14, 141.25, 139.65, 130.90, 130.87, 129.11, 128.88, 128.04, 126.87, 126.77, 125.72, 61.27, 44.47, 41.85; C_23_H_20_N_2_O_3_S: *m*/*z* (404.12).

##### 4.1.1.2. (5-(4-Chlorophenyl)-3-phenyl-4,5-dihydro-1*H*-pyrazol-1-yl)(4-(methylsulfonyl)phenyl)methanone (18b)

M.P. 321–323 °C, 75% yield (CH_3_OH); IR (KBr, cm^−1^) *ν*: 1654 (C=O), 1630 (C=N), 1369, 1220 (O=S=O); ^1^H NMR (700 MHz, DMSO) *δ* 8.16, 8.06 (d, *J* = 7.4, 8.3 Hz, 2*H*), 8.09 (d, *J* = 7.5 Hz, 2*H*), 7.74 (d, *J* = 7.7 Hz, 2*H*), 7.51–7.43 (m, 5*H*), 7.39 (d, *J* = 6.7 Hz, 2*H*), 5.82 (dd, *J* = 11.8, 5.0 Hz, 1*H*), 3.97 (dd, *J* = 18.1, 11.8 Hz, 1*H*), 3.34, 3.31 (s, 3*H*), 3.26 (dd, *J* = 17.9, 5.4 Hz, 1*H*); ^13^C NMR (176 MHz, DMSO) *δ* 164.58, 156.83, 142.89, 141.33, 139.59, 132.46, 131.21, 131.12, 130.70, 129.31, 129.26, 128.20, 127.44, 127.03, 60.57, 43.77, 42.02; C_23_H_19_ClN_2_O_3_S: *m*/*z* (438.00).

##### 4.1.1.3. (5-(4-Fluorophenyl)-3-phenyl-4,5-dihydro-1*H*-pyrazol-1-yl)(4-(methylsulfonyl)phenyl)methanone (18c)

M.P. 281–283 °C, 86% yield (CH_3_OH); IR (KBr, cm^−1^) *ν*: 1648 (C=O), 1311, 1146 (O=S=O); ^1^H NMR (700 MHz, DMSO) *δ* 8.12–8.06 (m, 4*H*), 7.75 (d, *J* = 4.7 Hz, 2*H*), 7.48 (d, *J* = 10.9 Hz, 3*H*), 7.41 (d, *J* = 8.9 Hz, 2*H*), 7.21 (t, *J* = 7.6 Hz, 2*H*), 5.82 (d, *J* = 10.8 Hz, 1*H*), 3.99–3.94 (m, 1*H*), 3.32 (s, 3*H*), 3.26 (d, *J* = 18.2 Hz, 1*H*); ^13^C NMR (176 MHz, DMSO) *δ* 164.58, 162.58, 161.20, 156.80, 142.86, 139.68, 138.60, 138.58, 131.19, 131.17, 130.70, 129.31, 128.98, 128.33, 128.29, 127.83, 127.43, 127.02, 116.10, 115.98, 60.49, 43.78, 42.14; C_23_H_19_FN_2_O_3_S: *m*/*z* (422.20).

##### 4.1.1.4. (3-(4-Fluorophenyl)-5-phenyl-4,5-dihydro-1*H*-pyrazol-1-yl)(4-(methylsulfonyl)phenyl)methanone (18d)

M.P. 311–313 °C, 82% yield (CH_3_OH); *ν*: 1648 (C=O), 1607 (C=N), 1289, 1148 (O=S=O); ^1^*H* NMR (500 MHz, CDCl_3_) *δ* 8.16 (d, *J* = 8.0 Hz, 2*H*), 8.05 (d, *J* = 8.0 Hz, 2*H*), 7.71 (dd, *J* = 8.6, 5.2 Hz, 2*H*), 7.40–7.33 (m, 5*H*), 7.14 (t, *J* = 10.0 Hz, 2*H*), 5.85 (dd, *J* = 11.9, 4.8 Hz, 1*H*), 3.85 (dd, *J* = 17.7, 11.8 Hz, 1*H*), 3.28 (dd, *J* = 17.7, 4.8 Hz, 1*H*), 3.12 (s, 3*H*); ^13^C NMR (126 MHz, CDCl_3_) *δ* 164.69, 163.25, 154.87, 142.18, 141.12, 130.82, 129.14, 128.94, 128.87, 128.09, 126.79, 125.68, 116.18, 116.01, 96.29, 61.33, 44.46, 41.92; C_23_H_19_FN_2_O_3_S: *m*/*z* (421.41).

##### 4.1.1.5. (3,5-Bis(4-fluorophenyl)-4,5-dihydro-1*H*-pyrazol-1-yl)(4-(methylsulfonyl)phenyl)methanone (18e)

M.P. 330–333 °C, 88% yield (CH_3_OH); IR (KBr, cm^−1^) *ν*: 1641 (C=O), 1608 (C=N), 1304, 1178 (O=S=O); ^1^H NMR (700 MHz, DMSO) *δ* 8.16 (d, *J* = 7.9 Hz, 1*H*-rotamter), 8.11 (d, *J* = 7.1 Hz, 1*H*-rotamer), 8.08 (d, *J* = 8.5 Hz, 2*H*-rotamer), 8.05 (d, *J* = 8.1 Hz, 2*H*-rotamer), 7.80 (t, *J* = 7.0 Hz, 2*H*), 7.40 (t, *J* = 7.0 Hz, 2*H*), 7.31 (t, *J* = 7.0 Hz, 2*H*), 7.21 (t, *J* = 8.6 Hz, 2*H*), 5.82 (dd, *J* = 11.8, 5.0 Hz, 1*H*), 3.96 (dd, *J* = 18.1, 11.8 Hz, 1*H*), 3.31 (s, 4.5*H*-rotamer), 3.28 (d, *J* = 5.1 Hz, 1.5*H*-rotamer); ^13^C NMR (176 MHz, DMSO) *δ* 165.12, 164.61, 163.21, 162.59, 161.21, 155.90, 144.07, 142.87, 139.67, 138.52, 137.30, 130.67, 129.88, 129.84, 128.99, 128.35, 128.31, 128.06, 127.82, 127.01, 116.47, 116.34, 116.09, 115.97, 60.60, 43.79, 43.72, 42.21; C_23_H_18_F_2_N_2_O_3_S: *m*/*z* (441.40).

##### 4.1.1.6. (4-(Methylsulfonyl)phenyl)(3-phenyl-5-(4-tolyl)-4,5-dihydro-1*H*-pyrazol-1-yl)methanone (18f)

M.P. 225–227 °C, 89% yield (C_2_H_5_OH); IR (KBr, cm^−1^) *ν*: 1629 (C=O), 1300, 1143 (O=S=O); ^1^H NMR (700 MHz, DMSO) *δ* 8.07 (q, *J* = 7.6 Hz, 4*H*), 7.73 (d, *J* = 7.4 Hz, 2*H*), 7.47 (q, *J* = 8.8 Hz, 3*H*), 7.22 (d, *J* = 7.9 Hz, 2*H*), 7.18 (d, *J* = 7.7 Hz, 2*H*), 5.76 (dd, *J* = 11.9, 4.7 Hz, 1*H*), 3.95 (dd, *J* = 18.1, 11.7 Hz, 1*H*), 3.30 (s, 3*H*), 3.21 (dd, *J* = 18.1, 4.8 Hz, 1*H*), 2.28 (s, 3*H*); ^13^C NMR (176 MHz, DMSO) *δ* 164.51, 156.81, 142.76, 139.84, 139.46, 137.16, 131.22, 131.15, 130.65, 129.82, 129.32, 128.99, 127.81, 127.37, 127.02, 126.01, 60.89, 43.79, 42.22, 21.12; (C_24_H_22_N_2_O_3_S): *m*/*z* (417.10).

##### 4.1.1.7. (3,5-Bis(4-tolyl)-4,5-dihydro-1*H*-pyrazol-1-yl)(4-(methylsulfonyl)phenyl)methanone (18g)

M.P. 190–192 °C, 83% yield (C_2_H_5_OH); IR (KBr, cm^−1^) *ν*: 1643 (C=O), 1298, 1142 (O=S=O); ^1^H NMR (500 MHz, CDCl_3_) *δ* 8.18 (d, *J* = 8.0 Hz, 2*H*), 8.04 (d, *J* = 8.0 Hz, 2*H*), 7.60 (d, *J* = 7.7 Hz, 2*H*), 7.25 (d, *J* = 7.4 Hz, 4*H*), 7.19 (d, *J* = 7.6 Hz, 2*H*), 5.79 (dd, *J* = 11.7, 4.9 Hz, 1*H*), 3.82 (dd, *J* = 17.6, 11.8 Hz, 1*H*), 3.26 (dd, *J* = 17.6, 4.8 Hz, 1*H*), 3.12 (s, 3*H*), 2.42 (s, 3*H*), 2.35 (s, 3*H*); ^13^C NMR (126 MHz, CDCl_3_) *δ* 164.45, 156.06, 142.01, 141.29, 139.82, 138.44, 137.72, 130.92, 129.72, 129.56, 129.31, 128.16, 126.83, 126.72, 125.70, 61.01, 44.47, 41.87, 21.56, 21.14; C_25_H_24_N_2_O_3_S: *m*/*z* (432.09).

##### 4.1.1.8. (5-(2-Hydroxyphenyl)-3-phenyl-4,5-dihydro-1*H*-pyrazol-1-yl)(4-(methylsulfonyl)phenyl)methanone (18h)

M.P. 343–345 °C, 76% yield (C_2_H_5_OH); ^1^H NMR (500 MHz, DMSO) *δ* 11.11 (s, 1*H*), 8.06 (m, 6*H*), 7.57 (m, 2*H*), 7.46 (t, *J* = 7.3 Hz, 2*H*), 7.40, (m, 3*H*), 5.29 (dd, *J* = 12.4, 2.9 Hz, 1*H*), 3.50 (dd, *J* = 17.1, 3.0 Hz, 1*H*), 3.27 (s, 3*H*), 2.91 (dd, *J* = 17.0, 12.4 Hz, 1*H*); ^13^C NMR (176 MHz, DMSO) *δ* 164.65, 163.45, 157.81, 157.63, 157.24, 150.57, 143.50, 142.88, 142.25, 140.20, 139.96, 139.03, 132.69, 132.34, 130.11, 129.70, 129.46, 129.34, 128.99, 128.89, 128.00, 127.40, 127.20, 127.08, 126.10, 125.29, 122.04, 120.41, 120.09, 118.20, 116.99, 116.53, 60.01, 44.14, 43.77; C_23_H_20_N_2_O_4_S: *m*/*z* (420.20).

##### 4.1.1.9. (5-(2-Methoxyphenyl)-3-phenyl-4,5-dihydro-1*H*-pyrazol-1-yl)(4-(methylsulfonyl)phenyl)methanone (18i)

M.P. 236–238 °C, 74% yield (C_2_H_5_OH); IR (KBr, cm^−1^) *ν*: 1639 (C=O), 1291, 1141 (O=S=O); ^1^H NMR (700 MHz, DMSO) *δ* 8.11 (t, *J* = 7.0 Hz, 2*H*), 8.07 (d, *J* = 8.5 Hz, 2*H*-rotamer), 7.72 (d, *J* = 6.5 Hz, 2*H*), 7.46 (dd, *J* = 12.0, 7.3 Hz, 3*H*), 7.30 (t, *J* = 7.0 Hz, 1*H*), 7.12 (d, *J* = 1.7 Hz, 1*H*), 7.09 (d, *J* = 8.2 Hz, 1*H*), 6.93 (t, *J* = 7.5 Hz, 1*H*), 5.93 (dd, *J* = 11.8, 4.8 Hz, 1*H*), 3.92 (dd, *J* = 17.8, 11.8 Hz, 1*H*), 3.86 (s, 3*H*), 3.32 (s, 3*H*), 3.10 (dd, *J* = 17.8, 4.8 Hz, 1*H*-rotamer); ^13^C NMR (176 MHz, DMSO) *δ* 165.11, 164.39, 157.23, 156.50, 144.07, 142.77, 139.90, 131.36, 131.05, 130.69, 129.34, 129.28, 129.25, 129.20, 128.99, 127.83, 127.31, 127.03, 126.19, 120.96, 111.97, 57.02, 56.12, 43.79, 41.14; C_24_H_22_N_2_O_4_S: *m*/*z* (434.20).

##### 4.1.1.10. (5-(4-Methoxyphenyl)-3-phenyl-4,5-dihydro-1*H*-pyrazol-1-yl)(4-(methylsulfonyl)phenyl)methanone (18j)

M.P. 258–260 °C, 84% yield (C_2_H_5_OH); IR (KBr, cm^−1^) *ν*: 1629 (C=O), 1299, 1145 (O=S=O); ^1^H NMR (700 MHz, DMSO) *δ* 8.07 (t, *J* = 7.0 Hz, 4*H*), 7.75 (d, *J* = 7.0 Hz, 2*H*), 7.47 (d, *J* = 12.8 Hz, 3*H*), 7.27 (t, *J* = 7.0 Hz, 2*H*), 6.94 (d, *J* = 7.1 Hz, 2*H*), 5.76 (t, *J* = 7.0 Hz, 1*H*), 3.94 (t, *J* = 14.8 Hz, 1*H*), 3.75 (s, 3*H*), 3.31 (s, 3*H*), 3.23 (d, *J* = 18.8 Hz, 1*H*); ^13^C NMR (176 MHz, DMSO) *δ* 164.49, 159.06, 156.80, 142.78, 139.88, 134.43, 131.28, 131.12, 130.64, 129.31, 127.46, 127.38, 127.01, 114.61, 60.62, 55.58, 43.80, 42.18; C_24_H_22_N_2_O_4_S: *m*/*z* (434.01).

##### 4.1.1.11. (3-(4-Methoxyphenyl)-5-phenyl-4,5-dihydro-1*H*-pyrazol-1-yl)(4-(methylsulfonyl)phenyl)methanone (18k)

M.P. 226–228 °C, 90% yield (C_2_H_5_OH); IR (KBr, cm^−1^) *ν*: 1636 (C=O), 1291, 1140 (O=S=O); ^1^H NMR (700 MHz, DMSO) *δ* 8.06 (t, *J* = 7.0 Hz, 4*H*), 7.74 (d, *J* = 7.2 Hz, 2*H*), 7.49–7.45 (m, 3*H*), 7.27 (d, *J* = 8.2 Hz, 2*H*), 6.93 (d, *J* = 8.2 Hz, 2*H*), 5.76 (dd, *J* = 11.6, 4.7 Hz, 1*H*), 3.94 (dd, *J* = 18.0, 11.7 Hz, 1*H*), 3.74 (s, 3*H*), 3.31 (s, 4.5*H*-rotamer), 3.23 (dd, *J* = 18.1, 4.7 Hz, 1.5*H*-rotamer); ^13^C NMR (176 MHz, DMSO) *δ* 164.47, 159.04, 156.80, 142.77, 139.86, 134.43, 131.27, 131.13, 130.63, 129.31, 127.81, 127.46, 127.38, 127.01, 114.60, 60.59, 55.57, 43.78, 42.18; C_24_H_22_N_2_O_4_S: *m*/*z* (434.10).

##### 4.1.1.12. (3,5-Bis(4-methoxyphenyl)-4,5-dihydro-1*H*-pyrazol-1-yl)(4-(methylsulfonyl)phenyl)methanone (18l)

M.P. 198–200 °C, 89% yield (C_2_H_5_OH); IR (KBr, cm^−1^) *ν*: 1640 (C=O), 1607 (C=N), 1246, 1143 (O=S=O); ^1^H NMR (500 MHz, CDCl_3_) *δ* 8.17 (d, *J* = 8.0 Hz, 2*H*), 8.03 (d, *J* = 8.0 Hz, 2*H*), 7.66 (d, *J* = 8.4 Hz, 2*H*), 7.29 (d, *J* = 7.6 Hz, 2*H*), 6.96 (d, *J* = 10.0 Hz, 2*H*), 6.90 (d, *J* = 5.0 Hz, 2*H*), 5.77 (dd, *J* = 11.8, 4.7 Hz, 1*H*), 3.88 (s, 3*H*), 3.81 (s, 3*H*), 3.73 (t, *J* = 7.0 Hz, 1*H*), 3.25 (dd, *J* = 17.6, 4.6 Hz, 1*H*), 3.11 (s, 3*H*); ^13^C NMR (126 MHz, CDCl_3_) *δ* 164.35, 161.73, 159.26, 155.75, 141.96, 139.91, 133.56, 130.88, 128.52, 127.13, 126.69, 123.53, 114.40, 114.29, 60.66, 55.45, 55.31, 44.47, 41.82; C_25_H_24_N_2_O_5_S: *m*/*z* (465.12).

##### 4.1.1.13. (5-(Benzo[*d*][1,3]dioxol-5-yl)-3-phenyl-4,5-dihydro-1*H*-pyrazol-1-yl)(4-(methylsulfonyl)phenyl)methanone (18m)

M.P. 155–157 °C, 76% yield (CH_3_CH_2_OH); IR (KBr, cm^−1^) *ν*: 1685 (C=O), 1638 (C=N), 1302, 1145 (O=S=O); ^1^H NMR (700 MHz, DMSO) *δ* 8.16 (d, *J* = 7.3 Hz, 1*H*), 8.10–8.04 (m, 3*H*), 7.74 (d, *J* = 7.3 Hz, 2*H*), 7.68 (d, *J* = 9.0 Hz, 1*H*), 7.58 (t, *J* = 6.6 Hz, 1*H*), 7.50–7.45 (m, 2*H*), 6.90 (d, *J* = 8.2 Hz, 1*H*), 6.83 (d, *J* = 8.1 Hz, 1*H*), 6.13, 6.01 (s, 2*H*-rotamer), 5.76–5.70 (m, 1*H*), 3.93 (dd, *J* = 17.1, 12.6 Hz, 1*H*), 3.34, 3.31 (s, 3*H*-rotamer), 3.26–3.21 (m, 1*H*); ^13^C NMR (176 MHz, DMSO) *δ* 164.55, 156.75, 150.09, 148.60, 148.08, 147.00, 144.58, 142.81, 139.80, 138.21, 136.34, 133.50, 131.25, 131.12, 130.69, 129.67, 129.29, 129.23, 128.94, 127.40, 127.00, 126.55, 120.40, 119.47, 109.03, 108.86, 107.45, 106.68, 102.15, 101.53, 60.91, 43.78, 42.21; C_24_H_20_N_2_O_5_S: *m*/*z* (448.30)

##### 4.1.1.14. (3-(2,4-Dimethoxyphenyl)-5-phenyl-4,5-dihydro-1*H*-pyrazol-1-yl)(4-(methylsulfonyl)phenyl)methanone (18n)

M.P. 216–218 °C, 77% yield (C_2_H_5_OH); IR (KBr, cm^−1^) *ν*: 1674 (C=O), 1608 (C=N), 1330, 1149 (O=S=O); ^1^H NMR (700 MHz, DMSO) *δ* 8.11 (d, *J* = 7.9 Hz, 3*H*), 8.04 (d, *J* = 8.0 Hz, 2*H*), 7.65 (d, *J* = 8.7 Hz, 1*H*), 7.38 (t, *J* = 7.5 Hz, 2*H*), 7.29 (dd, *J* = 7.0 Hz, 2*H*), 5.72 (dd, *J* = 11.6, 4.5 Hz, 1*H*), 3.96 (dd, *J* = 18.4, 11.6 Hz, 1*H*), 3.81 (s, 6*H*), 3.31, 3.30 (s, 3*H*-rotamer), 3.18 (dd, *J* = 18.4, 4.5 Hz, 1*H*); ^13^C NMR (176 MHz, DMSO) *δ* 165.13, 164.05, 163.10, 160.09, 155.66, 144.04, 142.72, 142.66, 139.93, 137.29, 130.76, 130.64, 129.30, 128.99, 127.82, 126.91, 125.88, 112.64, 106.76, 99.12, 60.55, 56.31, 55.96, 45.34, 43.78, 43.70; C_25_H_24_N_2_O_5_S: *m*/*z* (465.00).

##### 4.1.1.15. (3-(2,4-Dimethoxyphenyl)-5-(4-methoxyphenyl)-4,5-dihydro-1*H*-pyrazol-1-yl)(4-(methylsulfonyl)phenyl)methanone (18o)

M.P. 268–270 °C, 95% yield (C_2_H_5_OH); IR (KBr, cm^−1^) *ν*: 1633 (C=O), 1301, 1146 (O=S=O); ^1^*H* NMR (500 MHz, CDCl_3_) *δ* 8.20 (d, *J* = 8.0 Hz, 2*H*), 8.01 (d, *J* = 8.1 Hz, 2*H*), 7.76 (d, *J* = 8.7 Hz, 1*H*), 7.30 (d, *J* = 10.0 Hz, 2*H*), 6.90 (d, *J* = 8.1 Hz, 2*H*), 6.56 (dd, *J* = 10.0, 5.0 Hz, 1*H*), 5.70 (dd, *J* = 11.5, 4.6 Hz, 1*H*), 3.92 (t, *J* = 5.0 Hz, 1*H*), 3.88 (s, 3*H*), 3.85 (s, 3*H*), 3.81 (s, 3*H*), 3.40 (dd, *J* = 18.6, 4.6 Hz, 1*H*), 3.10 (s, 3*H*); ^13^C NMR (126 MHz, CDCl_3_) *δ* 164.16, 163.04, 159.85, 159.12, 155.80, 141.81, 130.99, 130.46, 127.19, 126.62, 114.30, 105.64, 98.69, 60.49, 55.55, 55.51, 55.30, 44.89, 44.49; C_26_H_26_N_2_O_6_S: *m*/*z* (495.20).

##### 4.1.1.16. (3-(2,4-Dimethoxyphenyl)-5-(3,4-dimethoxyphenyl)-4,5-dihydro-1*H*-pyrazol-1-yl)(4-(methylsulfonyl)phenyl)methanone (18p)

M.P. 169–171 °C, 90% yield (C_2_H_5_OH); IR (KBr, cm^−1^) *ν*: 1636 (C=O), 1601 (C=N), 1295, 1145 (O=S=O); ^1^H NMR (700 MHz, DMSO) *δ* 8.09 (d, *J* = 8.1 Hz, 2*H*), 8.04 (d, *J* = 8.3 Hz, 2*H*), 7.63 (d, *J* = 8.6 Hz, 1*H*), 6.92 (t, *J* = 7.0 Hz, 2*H*), 6.79 (d, *J* = 8.2 Hz, 1*H*), 6.64 (s, 1*H*), 6.60 (d, *J* = 8.8 Hz, 1*H*), 5.66 (dd, *J* = 11.6, 4.5 Hz, 1*H*), 3.91 (dd, *J* = 18.4, 11.4 Hz, 1*H*), 3.82 (s3*H*), 3.81 (s, 3*H*), 3.75 (s, 3*H*), 3.73 (s, 3*H*), 3.30 (d, *J* = 11.4 Hz, 4*H*-rotamer), 3.20 (dd, *J* = 18.3, 4.6 Hz, 1*H*); ^13^C NMR (176 MHz, DMSO) *δ* 164.11, 163.06, 160.07, 155.76, 149.32, 148.50, 142.61, 140.11, 135.15, 130.68, 130.60, 128.99, 127.81, 126.91, 117.55, 112.80, 112.54, 110.04, 106.73, 99.15, 60.32, 56.31, 56.01, 55.95, 45.33, 43.79, 43.71; C_27_H_28_N_2_O_7_S: *m*/*z* (524.25).

##### 4.1.1.17. (3-(3,4-Dimethoxyphenyl)-5-(3,4,5-trimethoxyphenyl)-4,5-dihydro-1*H*-pyrazol-1-yl)(4-(methylsulfonyl)phenyl)methanone (18q)

M.P. 150–152 °C, 87% yield (C_2_H_5_OH); IR (KBr, cm^−1^) *ν*: 1647 (C=O), 1256, 1117 (O=S=O); ^1^H NMR (700 MHz, DMSO) *δ* 8.13 (d, *J* = 8.0 Hz, 2*H*), 8.07 (d, *J* = 8.0 Hz, 2*H*), 7.30 (d, *J* = 8.0 Hz, 1*H*), 7.25 (s, 1*H*), 7.02 (d, *J* = 8.4 Hz, 1*H*), 6.60 (s, 2*H*), 5.74 (dd, *J* = 11.7, 5.0 Hz, 1*H*), 3.89 (dd, *J* = 17.9, 11.6 Hz, 1*H*), 3.80 (s, 3*H*), 3.78 (s, 3*H*), 3.76 (s, 6*H*), 3.65 (s, 3*H*), 3.31 (s, 3*H*), 3.27 (d, *J* = 5.1 Hz, 1*H*). ^13^C NMR (176 MHz, DMSO) *δ* 164.39, 156.80, 153.64, 151.49, 149.14, 142.77, 139.93, 138.25, 137.02, 130.71, 127.02, 123.79, 121.05, 112.01, 110.25, 102.90, 61.21, 60.40, 56.34, 56.08, 55.99, 43.81, 42.44; C_28_H_30_N_2_O_8_S: *m*/*z* (554.90).

### 4.2. Biological evaluation

#### 4.2.1. *In vitro* antitumor assay

The antitumor experiment was conducted on 59 human tumor cell lines derived from nine different human tissues, following the procedure of the Drug Evaluation Branch at the NCI, Bethesda, MD.^33^ The MTT assay was carried out to assess the *in vitro* antitumor activity of the compounds 18c, 18g, and 18h, as per the documented procedure, utilizing leukemia HL-60, breast adenocarcinoma MCF-7, and MDA-MB-231 cancer cell lines.^77^ The cytotoxicity of the selected compounds 18c, 18g, and 18h against a normal WI-38 cell line was estimated according to the reported procedure.^33,77^

#### 4.2.2. *In vitro* COX-2 inhibition assay

The colorimetric COX-2 inhibition assay (kit catalogue number 560101, Cayman Chemical, Ann Arbour, MI) was employed to assess the inhibitory power of celecoxib and the evaluated derivatives on COX-2 isozyme, following the guidelines provided by the manufacturer's instructions.^33,60,61^

#### 4.2.3. *In vitro* EGFR, HER2, and VEGFR2 tyrosine kinases assay

The EGFR, HER2, and VEGFR2 enzyme inhibition assays were performed as described in the previous reports The EGFR, HER2, and VEGFR2 enzyme inhibition experiments were carried out according to the methods outlined in earlier papers, utilizing the most potent pyrazolines 18c, 18g, and 18h.^33,77^

#### 4.2.4. Apoptosis assay

As per our earlier study, we induced apoptosis using the leukemia HL-60 cell line and a widely recognized Annexin 5-FITC/PI detection kit. The cell line samples were examined utilizing a FACSCalibur flow cytometer.^33,77^

#### 4.2.5. Cell cycle analysis

The cell cycle analysis was conducted similarly to our prior study, using the leukemia HL-60 cell line. The cells were stained with the DNA fluorochrome PI and analyzed using a FACSCalibur flow cytometer.^33,77^

#### 4.2.6. Apoptotic markers assay

Apoptotic markers experiment was performed according to reported procedure using MDA-MB-231 cancer cell line.^78–80^

### 4.3. Molecular docking methodology

#### 4.3.1. Ligand preparation

Both enantiomers of compound 18h were drawn in Maestro using the available drawing tools. The structures were minimized using MacroModel (Macromodel release 2020-3, Schrödinger, LLC, New York, 2020) with the force field OPLS3. The solvent effects were simulated by the solvation model GB/SA with ‘‘no cutoff’’ for non-bonded interactions, and minimization was conducted by employing the PRCG method (5000 maximum iterations and 0.001 gradient convergence threshold). Finally, to generate the most probable ionization states at physiological pH (7.4 ± 0.2), the enantiomers were then treated with the LigPrep application (LigPrep release 2020-3, Schrödinger, LLC, New York, 2020).^81^

#### 4.3.2. Protein preparation

The target proteins were downloaded from the Protein Data Bank (PDB) and prepared using the Protein Preparation Wizard implemented in Maestro Suite (Maestro release 2020-3, Schrödinger, LLC, New York, 2020), as previously reported.^82^ In particular, we used human EGFR (PDB ID 1M17), human HER2 (PDB ID 3RCD), and human VEGFR2 (PDB ID 4ASD). We removed the materials utilized for the crystallisation process.

#### 4.3.3. Molecular docking

The software Glide (Glide release 2020-3, Schrödinger, LLC, New York, 2020) was utilized to conduct molecular docking studies on the selected targets by applying the Glide standard precision (SP) scoring function. Energy grids for all proteins were prepared using the default values of the protein atom scaling factor (1.0 Å) within a cubic box centred on the crystallized ligands. Both enantiomers of compound 18h were docked into the selected binding site without any constraint using default parameters. The number of docked poses entered for post-docking minimization was set to 100. The Glide SP score and a visual inspection of the selected docked pose belonging to the most populated cluster obtained from the molecular docking calculation were evaluated.

## Data availability

Data are available as ESI† (Data supporting this study are included within the article and ESI file†).

## Conflicts of interest

The authors declare that they have no conflict of interest.

## Supplementary Material

RA-014-D4RA03902E-s001
