# Supplementary material for: Synthesis, enzyme inhibition assay, and molecular modeling study of novel pyrazolines linked to 4-methylsulfonylphenyl scaffold: antitumor activity and cell cycle analysis
Source: RSC Adv. 2024 Jul 12;14(31):22132–46. doi: 10.1039/d4ra03902e (PMC11240878; doi:10.1039/d4ra03902e)
Supplement: RA-014-D4RA03902E-s001 [file RA-014-D4RA03902E-s001.pdf]

Electronic Supplementary Material (ESI) for RSC Advances.
This journal is © The Royal Society of Chemistry 2024

Synthesis, enzyme inhibition assay, and molecular modeling study of novel pyrazolines linked to 4-methylsulfonylphenyl scaffold: Antitumor activity and cell cycle analysis

Alaa A.-M. Abdel-Aziz, Adel S. El-Azab, Simone Brogi, , Rezk R. Ayyad*, Hamad M. Alkahtani,

Hatem A. Abuelizz, Ibrahim A. Al-Suwaidan, Abdulrahman M. Al-Obaid

NMR Figures

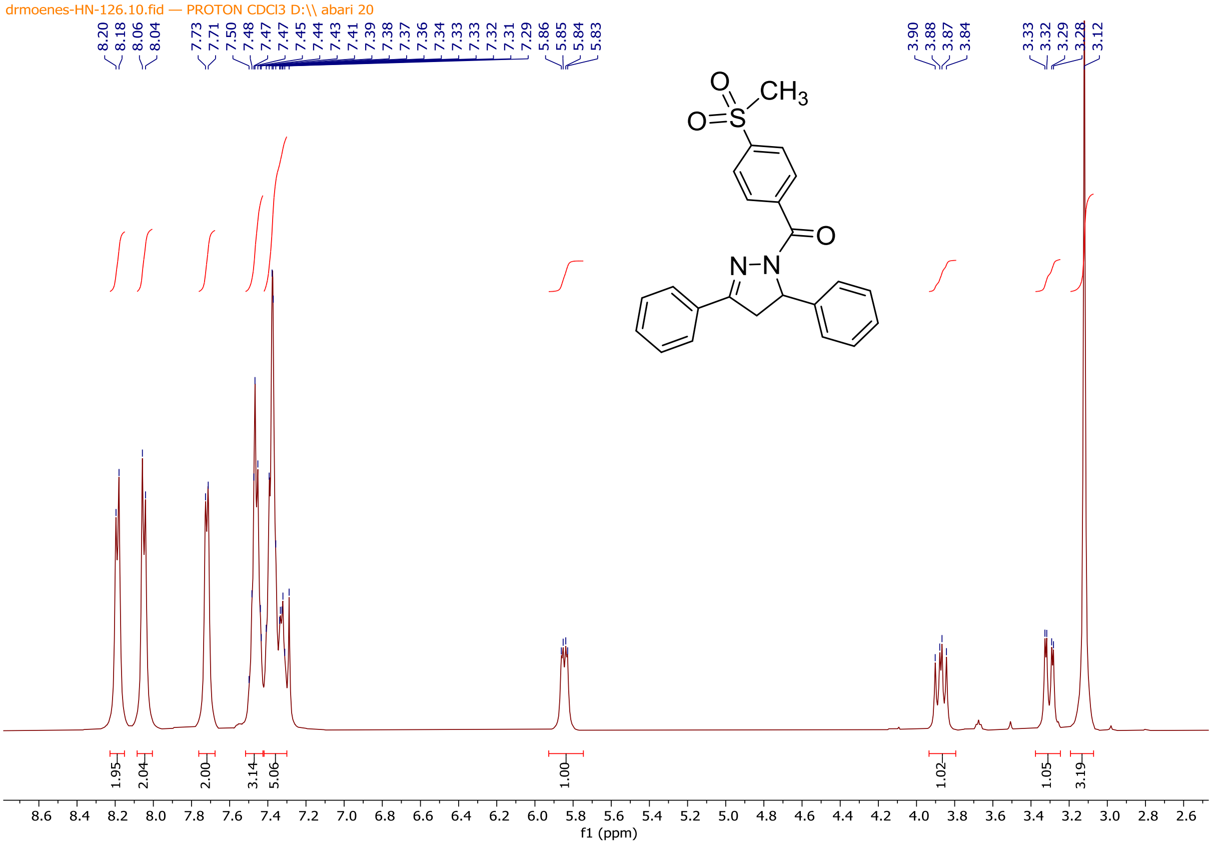
drmoenes-HN-126.10.fid — PROTON CDCl3 D:\\ abari 20
8.20 8.18 8.06 8.04 7.73 7.71 7.50 7.48 7.47 7.47 7.45 7.44 7.43 7.41 7.39 7.38 7.37 7.36 7.34 7.33 7.33 7.32 7.31 7.29 5.86 5.85 5.84 5.83 3.90 3.88 3.87 3.84 3.33 3.32 3.29 3.28 3.12
1.95 2.04 2.00 3.14 5.06 1.00 1.02 1.05 3.19
O
O
S
CH3
O
N
N
f1 (ppm)

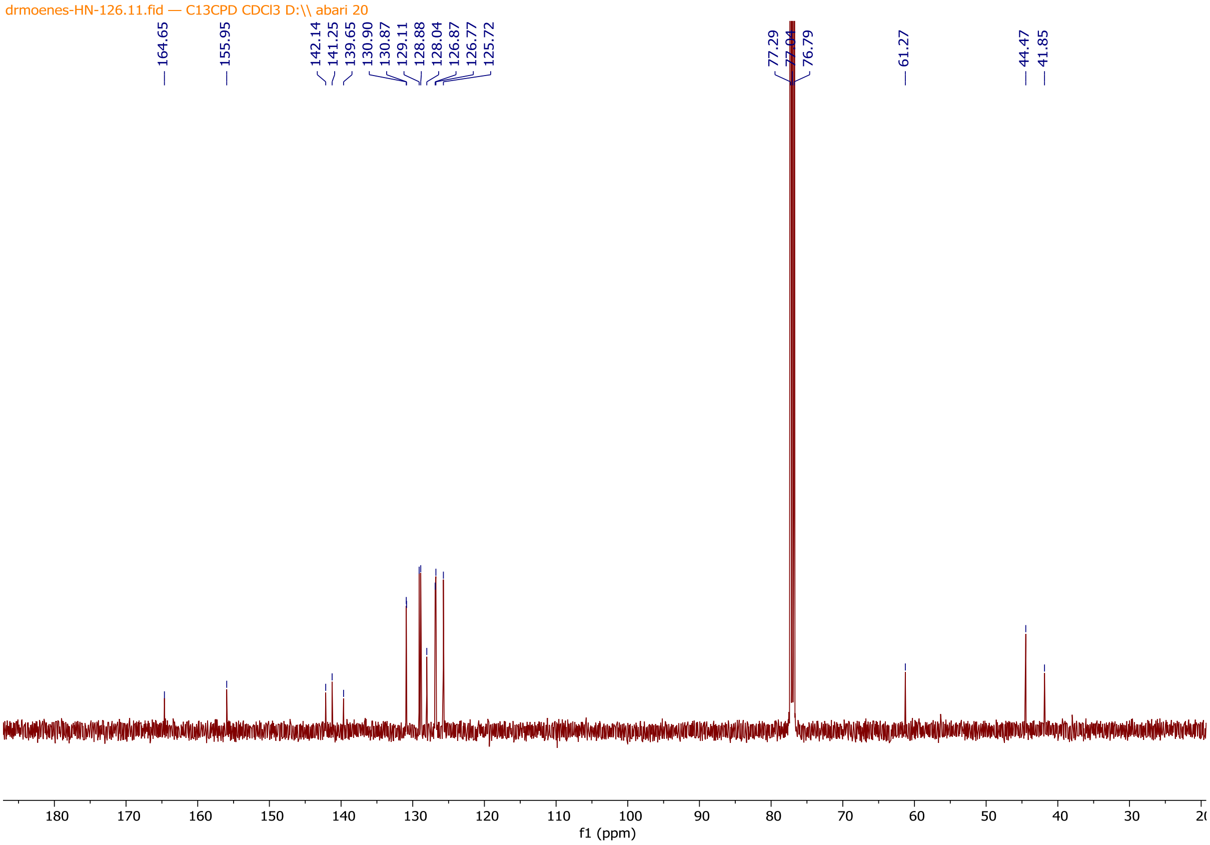
drmoenes-HN-126.11.fid — C13CPD CDCl3 D:\\ abari 20
164.65 155.95 142.14 141.25 139.65 130.90 130.87 129.11 128.88 128.04 126.87 126.77 125.72 77.29 77.04 76.79 61.27 44.47 41.85
f1 (ppm)

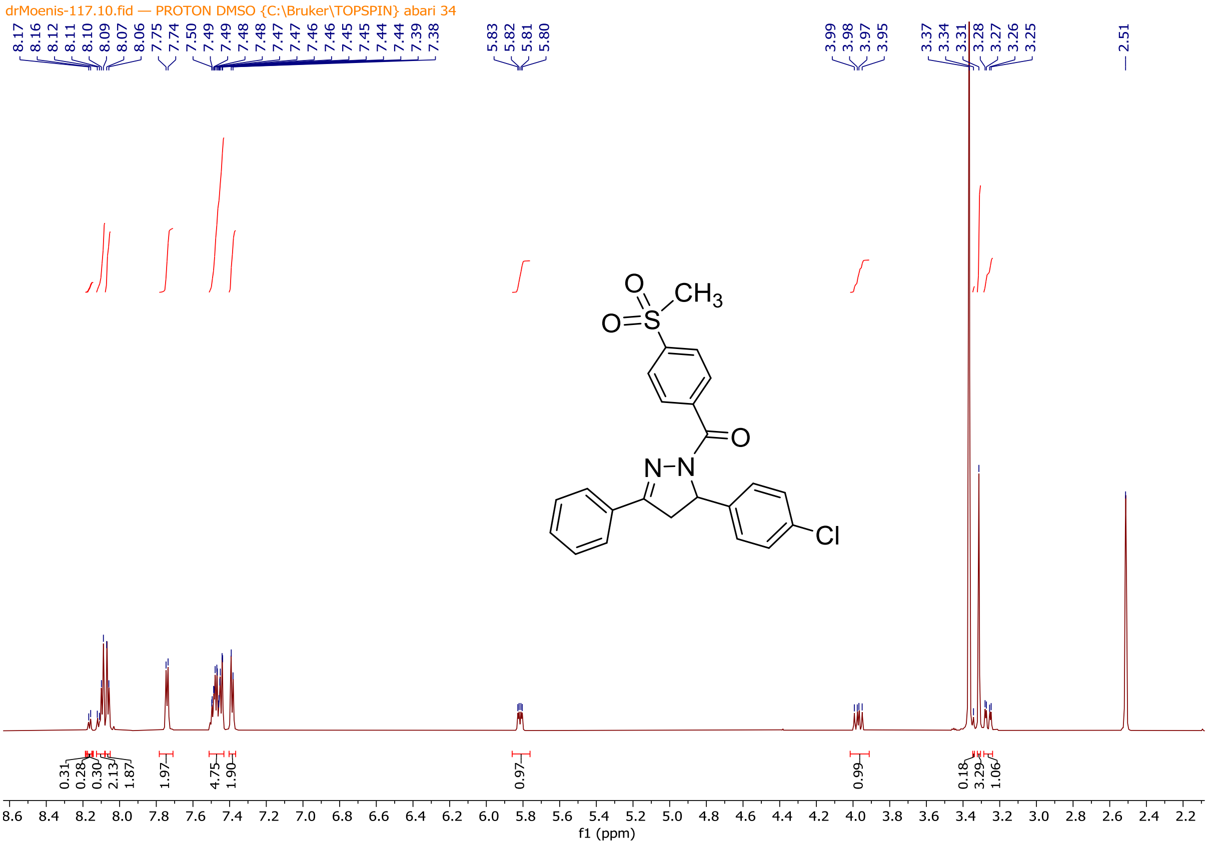
drMoenis-117.10.fid — PROTON DMSO {C:\Bruker\TOPSPIN} abari 34
8.17 8.16 8.12 8.11 8.10 8.09 8.07 8.06 7.75 7.74 7.50 7.49 7.49 7.48 7.48 7.47 7.47 7.46 7.46 7.45 7.45 7.44 7.44 7.39 7.38
5.83 5.82 5.81 5.80
3.99 3.98 3.97 3.95
3.37 3.34 3.31 3.28 3.27 3.26 3.25
2.51
CH3
O
O=S
O
N–N
Cl
0.31 0.28 0.30 2.13 1.87 1.97 4.75 1.90 0.97 0.99 0.18 3.29 1.06
f1 (ppm)

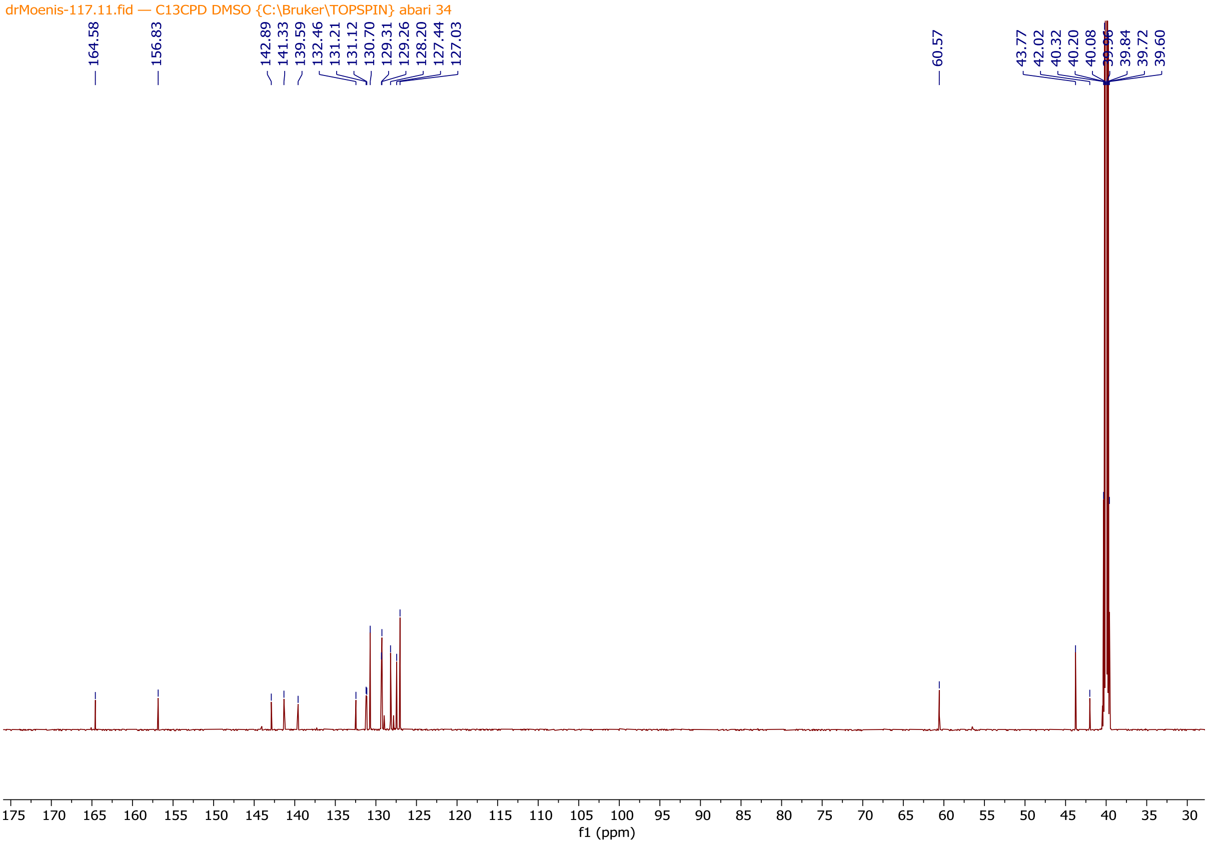
drMoenis-117.11.fid — C13CPD DMSO {C:\Bruker\TOPSPIN} abari 34
164.58
156.83
142.89 141.33 139.59 132.46 131.21 131.12 130.70 129.31 129.26 128.20 127.44 127.03
60.57
43.77 42.02 40.32 40.20 40.08 39.96 39.84 39.72 39.60
f1 (ppm)

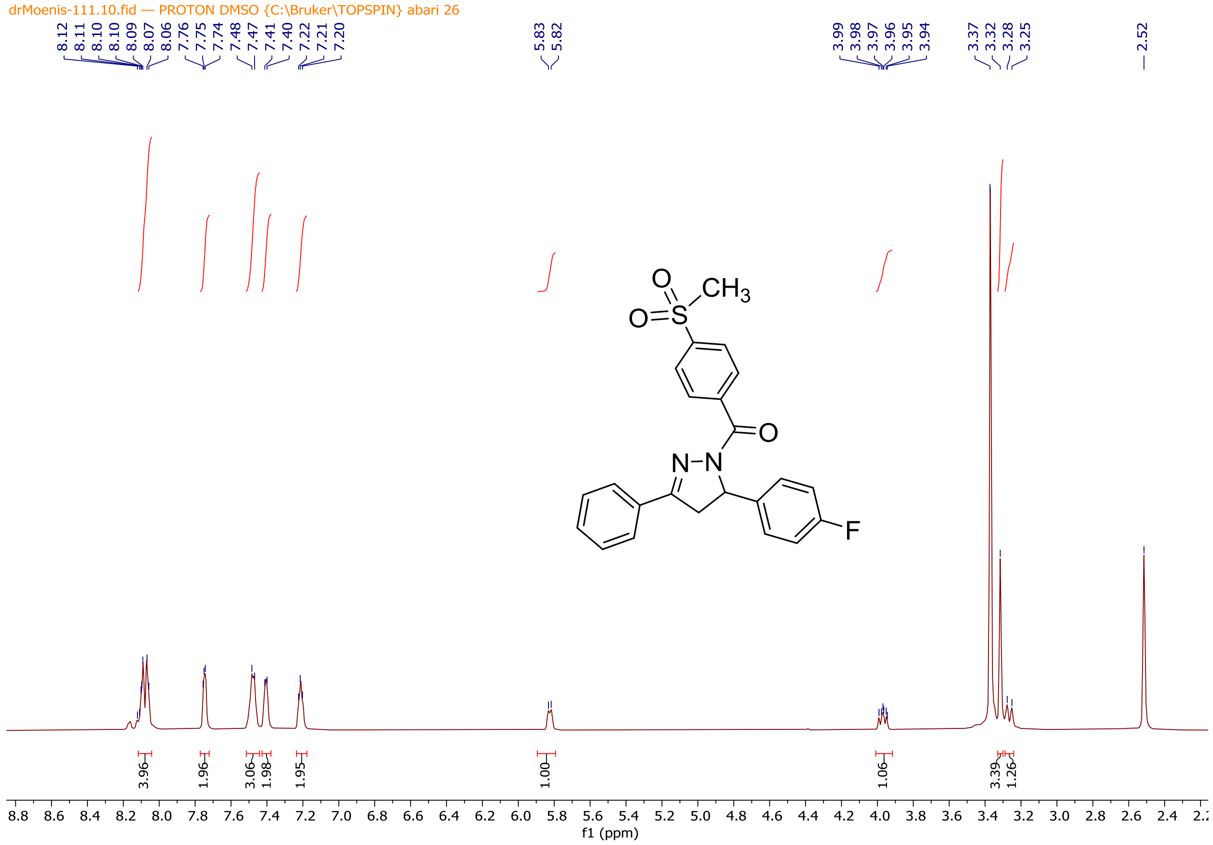
drMoenis-111.10.fid — PROTON DMSO {C:\Bruker\TOPSPIN} abari 26
8.12 8.11 8.10 8.10 8.09 8.07 8.06 7.76 7.75 7.74 7.48 7.47 7.41 7.40 7.22 7.21 7.20
5.83 5.82
3.99 3.98 3.97 3.96 3.95 3.94 3.37 3.32 3.28 3.25
2.52
O
CH3
O=S
O
N–N
F
3.96 1.96 3.06 1.98 1.95 1.00 1.06 3.39 1.26
f1 (ppm)

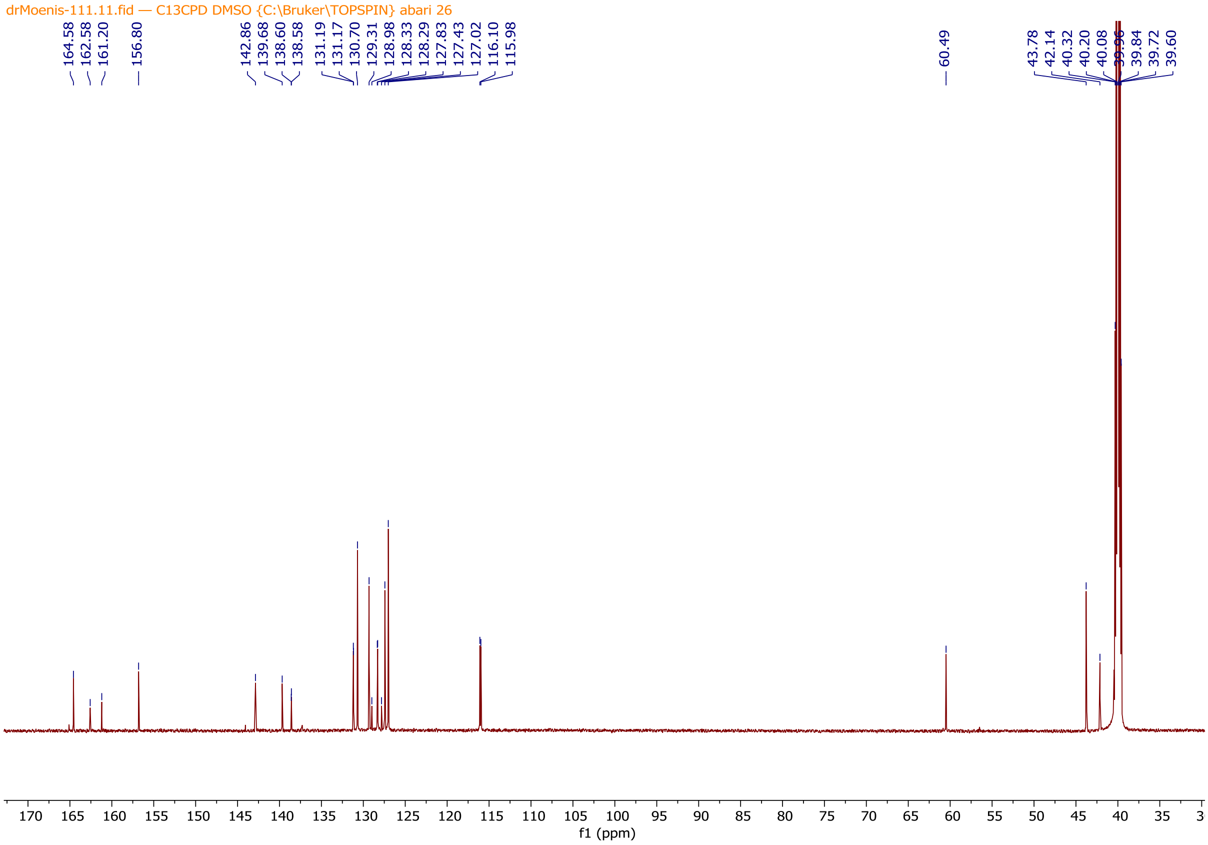
drMoenis-111.11.fid — C13CPD DMSO {C:\Bruker\TOPSPIN} abari 26
164.58 162.58 161.20 156.80
142.86 139.68 138.60 138.58 131.19 131.17 130.70 129.31 128.98 128.33 128.29 127.83 127.43 127.02 116.10 115.98
60.49
43.78 42.14 40.32 40.20 40.08 39.96 39.84 39.72 39.60
f1 (ppm)

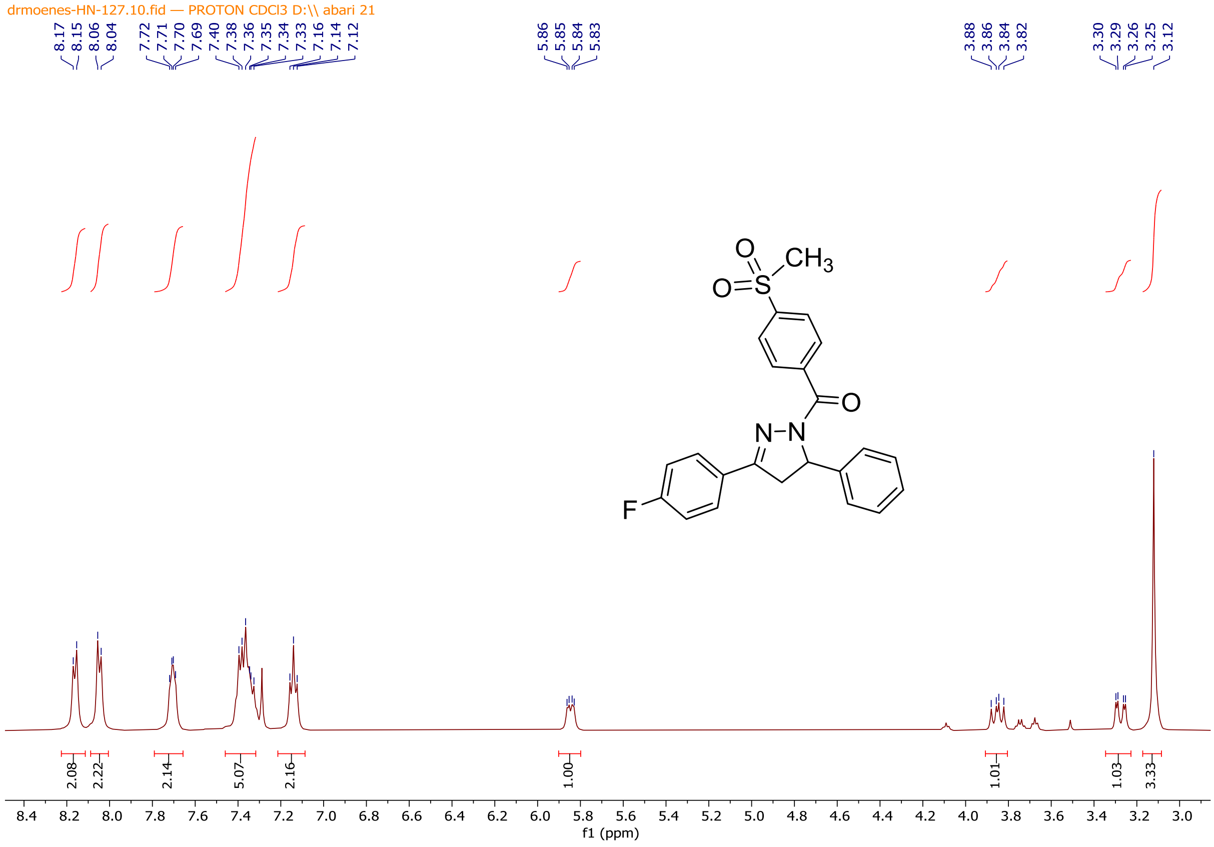
drmoenes-HN-127.10.fid — PROTON CDCl3 D:\\ abari 21
8.17 8.15 8.06 8.04 7.72 7.71 7.70 7.69 7.40 7.38 7.36 7.35 7.34 7.33 7.16 7.14 7.12
5.86 5.85 5.84 5.83
3.88 3.86 3.84 3.82
3.30 3.29 3.26 3.25 3.12
O
CH3
O=S
O
N-N
F
2.08 2.22 2.14 5.07 2.16 1.00 1.01 1.03 3.33
8.4 8.2 8.0 7.8 7.6 7.4 7.2 7.0 6.8 6.6 6.4 6.2 6.0 5.8 5.6 5.4 5.2 5.0 4.8 4.6 4.4 4.2 4.0 3.8 3.6 3.4 3.2 3.0
f1 (ppm)

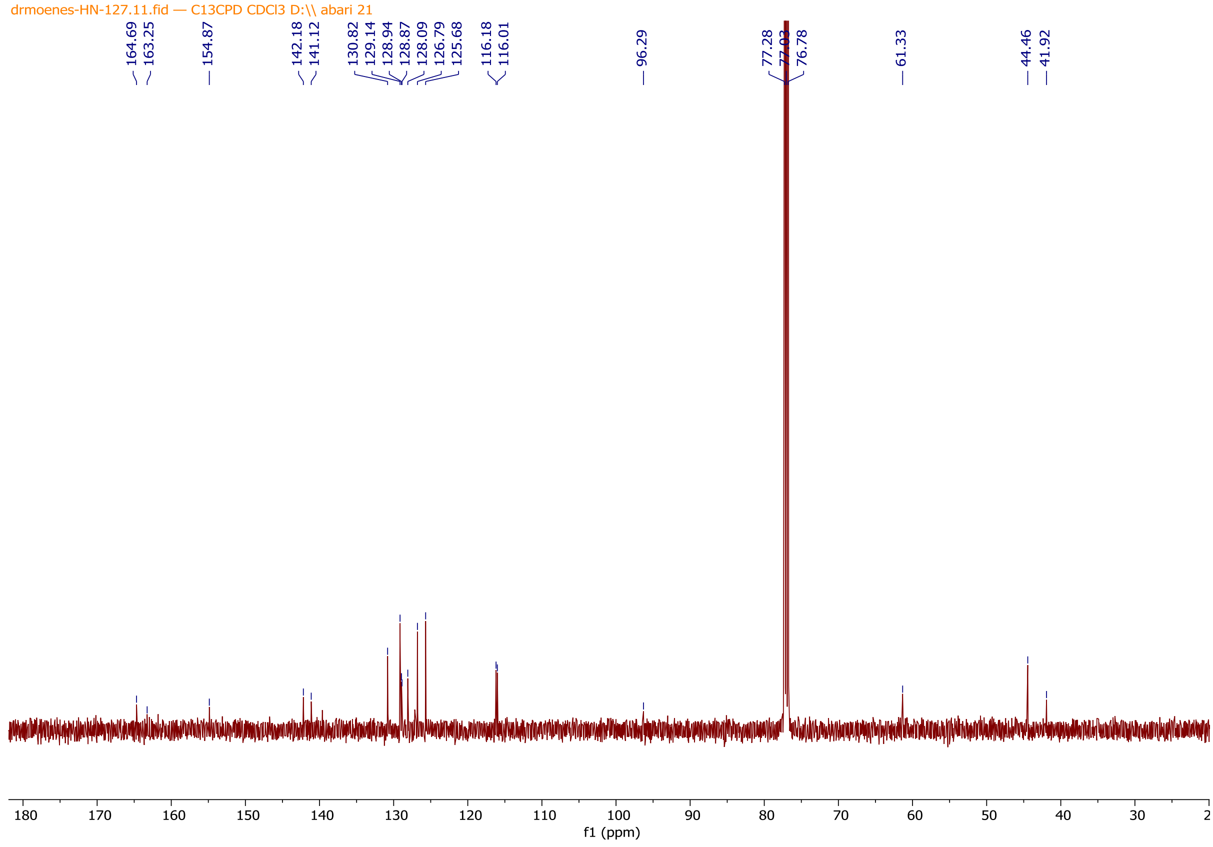
drmoenes-HN-127.11.fid — C13CPD CDCl3 D:\\ abari 21
164.69 163.25 154.87 142.18 141.12 130.82 129.14 128.94 128.87 128.09 126.79 125.68 116.18 116.01 96.29 77.28 77.03 76.78 61.33 44.46 41.92
180 170 160 150 140 130 120 110 100 90 80 70 60 50 40 30 2
f1 (ppm)

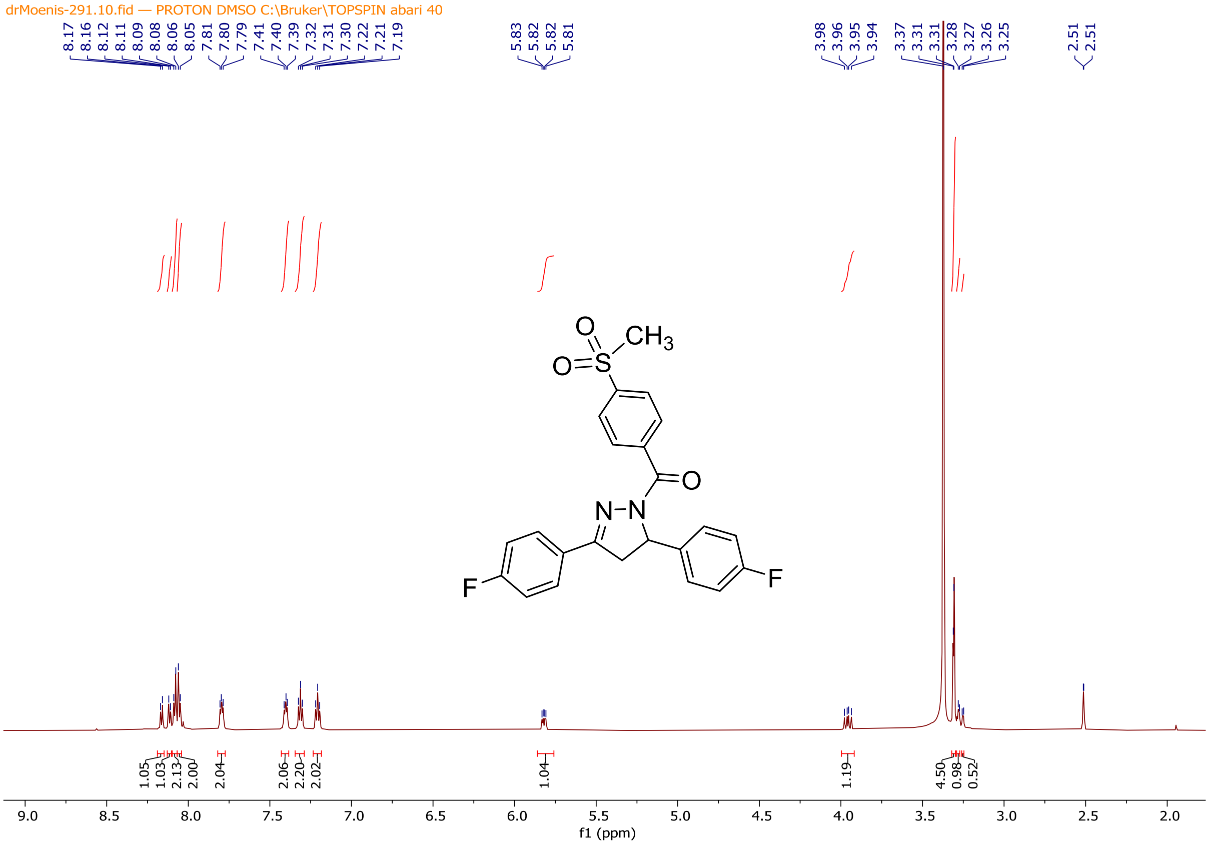
drMoenis-291.10.fid — PROTON DMSO C:\Bruker\TOPSPIN abari 40
8.17 8.16 8.12 8.11 8.09 8.08 8.06 8.05 7.81 7.80 7.79 7.41 7.40 7.39 7.32 7.31 7.30 7.22 7.21 7.19
5.83 5.82 5.82 5.81
3.98 3.96 3.95 3.94 3.37 3.31 3.31 3.28 3.27 3.26 3.25
2.51 2.51
O
CH3
O=S
O
N–N
F
F
1.05 1.03 2.13 2.00 2.04 2.06 2.20 2.02 1.04 1.19 4.50 0.98 0.52
9.0 8.5 8.0 7.5 7.0 6.5 6.0 5.5 5.0 4.5 4.0 3.5 3.0 2.5 2.0
f1 (ppm)

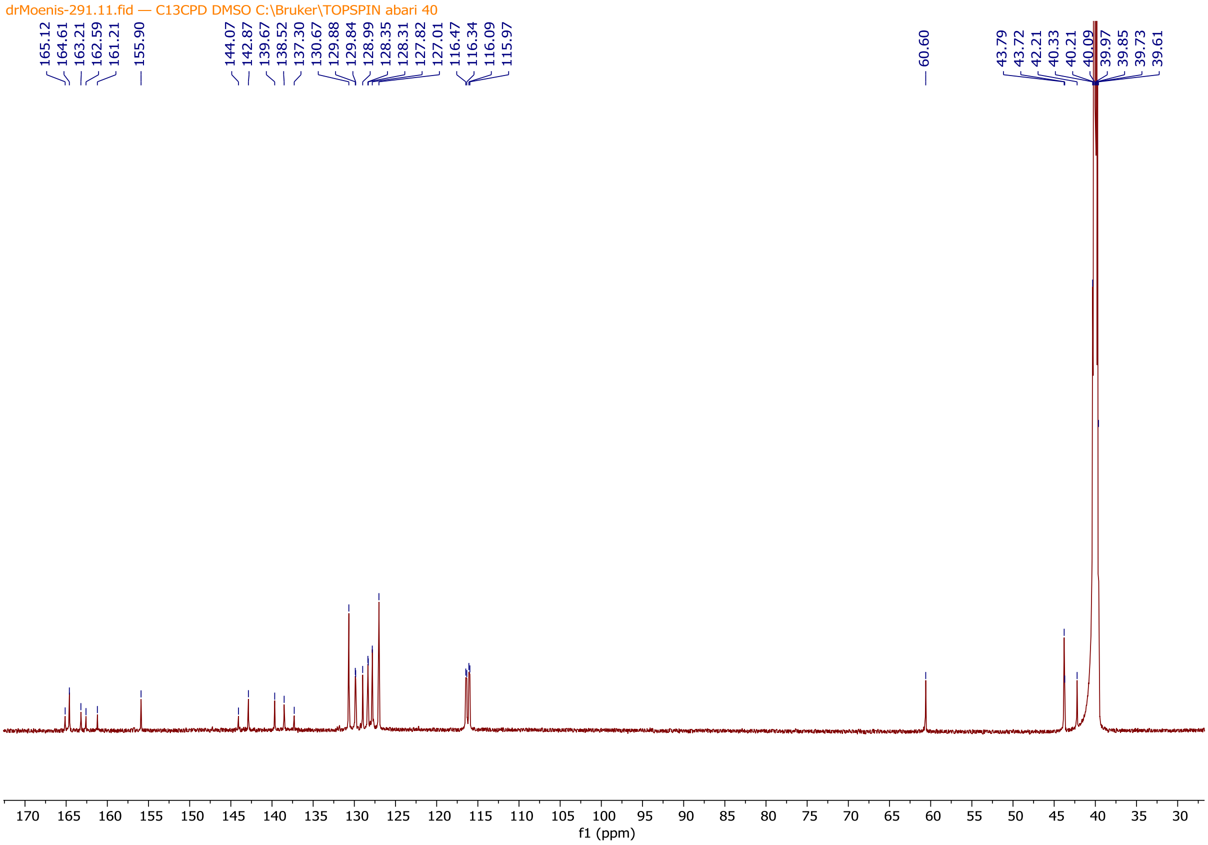
drMoenis-291.11.fid — C13CPD DMSO C:\Bruker\TOPSPIN abari 40
165.12 164.61 163.21 162.59 161.21 155.90
144.07 142.87 139.67 138.52 137.30 130.67 129.88 129.84 128.99 128.35 128.31 127.82 127.01 116.47 116.34 116.09 115.97
60.60
43.79 43.72 42.21 40.33 40.21 40.09 39.97 39.85 39.73 39.61
170 165 160 155 150 145 140 135 130 125 120 115 110 105 100 95 90 85 80 75 70 65 60 55 50 45 40 35 30
f1 (ppm)

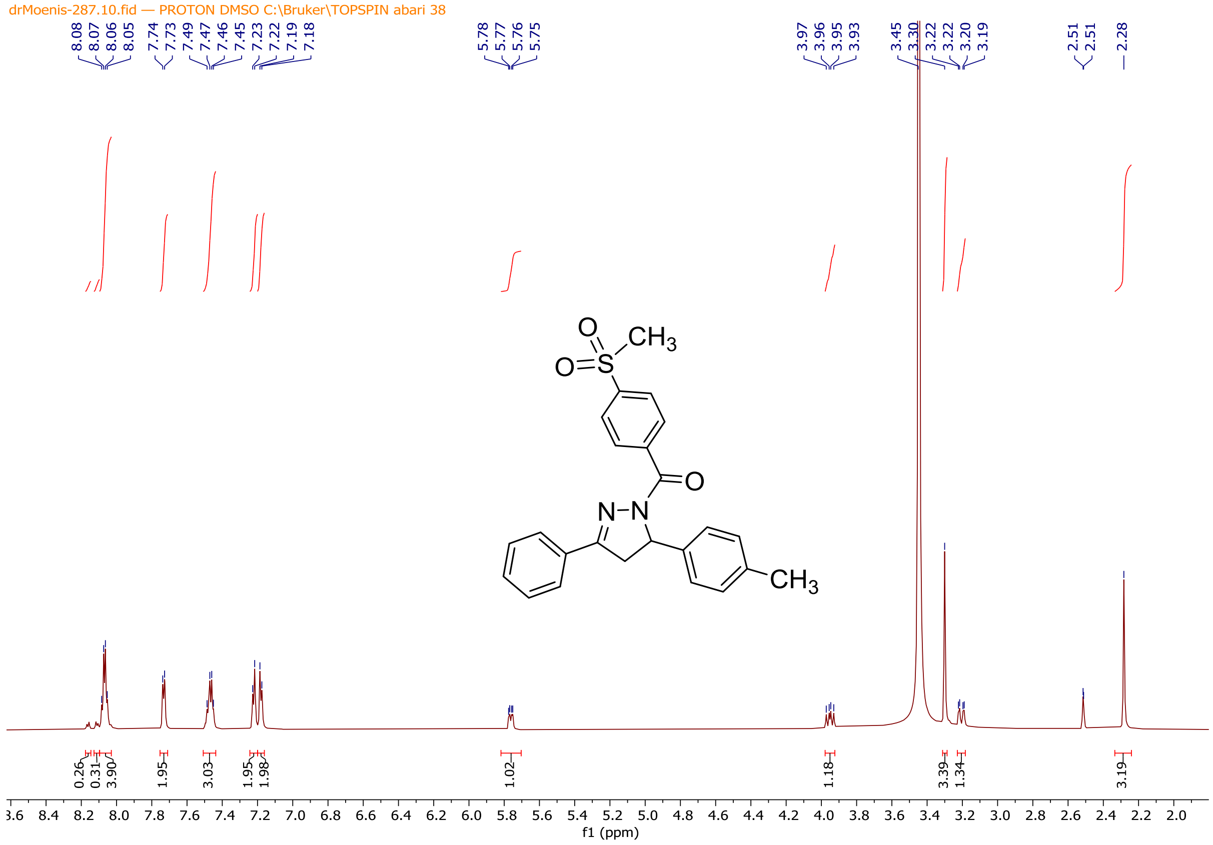
drMoenis-287.10.fid — PROTON DMSO C:\Bruker\TOPSPIN abari 38
8.08 8.07 8.06 8.05 7.74 7.73 7.49 7.47 7.46 7.45 7.23 7.22 7.19 7.18
5.78 5.77 5.76 5.75
3.97 3.96 3.95 3.93 3.45 3.30 3.22 3.22 3.20 3.19
2.51 2.51 2.28
0.26 0.31 3.90 1.95 3.03 1.95 1.98 1.02 1.18 3.39 1.34 3.19
O
CH3
O=S
O
N–N
CH3
f1 (ppm)

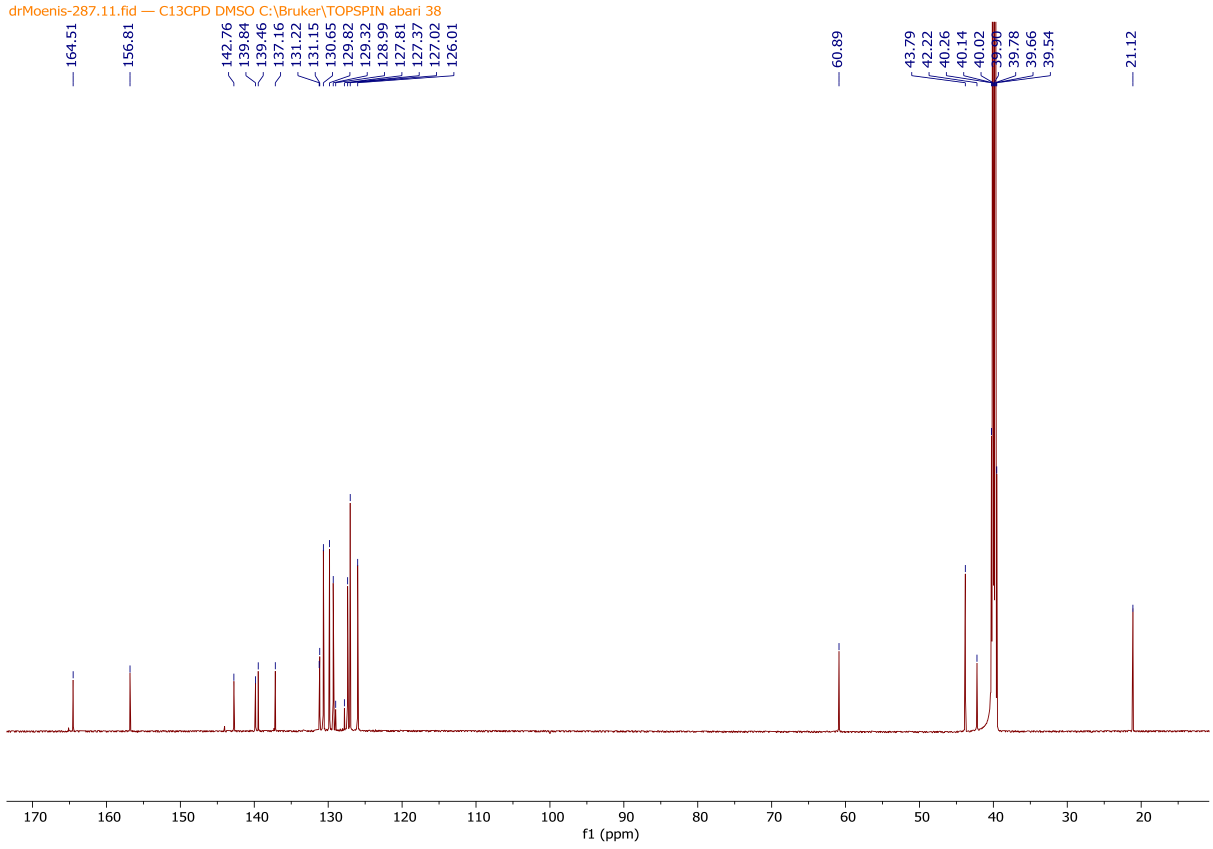
drMoenis-287.11.fid — C13CPD DMSO C:\Bruker\TOPSPIN abari 38
164.51
156.81
142.76 139.84 139.46 137.16 131.22 131.15 130.65 129.82 129.32 128.99 127.81 127.37 127.02 126.01
60.89
43.79 42.22 40.26 40.14 40.02 39.90 39.78 39.66 39.54
21.12
f1 (ppm)

drmoenes-HN-125.10.fid — PROTON CDCl3 D:\\ abari 5

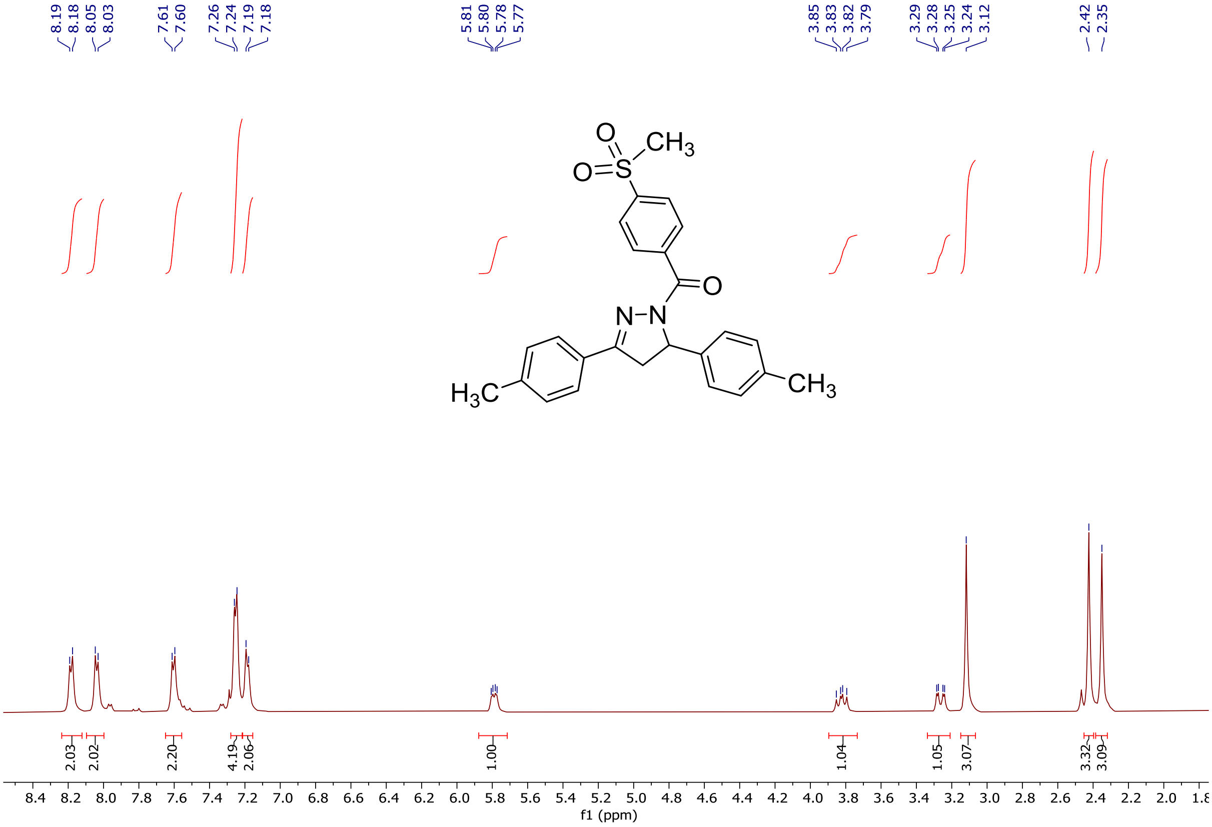

drmoenes-HN-125.11.fid — C13CPD CDCl3 D:\\ abari 5

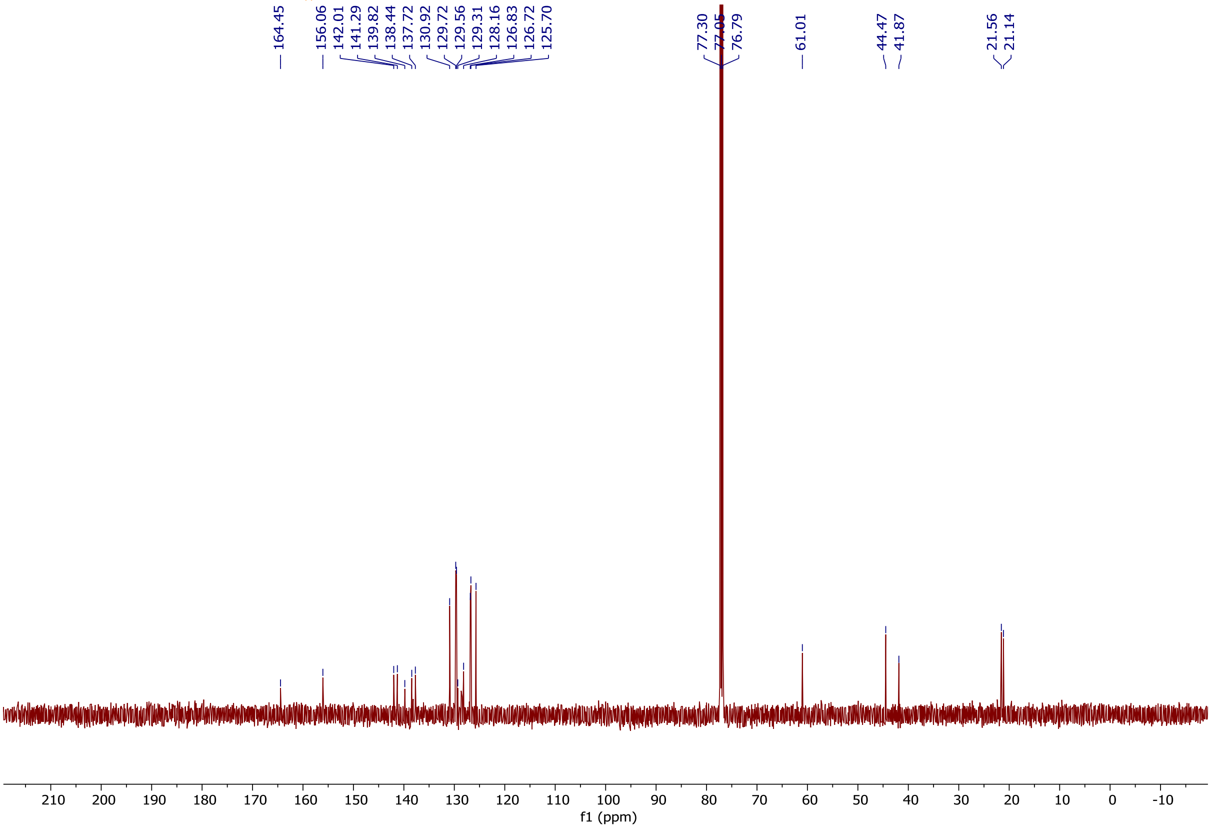

drmoenes-MS-24.10.fid — PROTON DMSO {C:\Bruker\TOPSPIN} abari 52

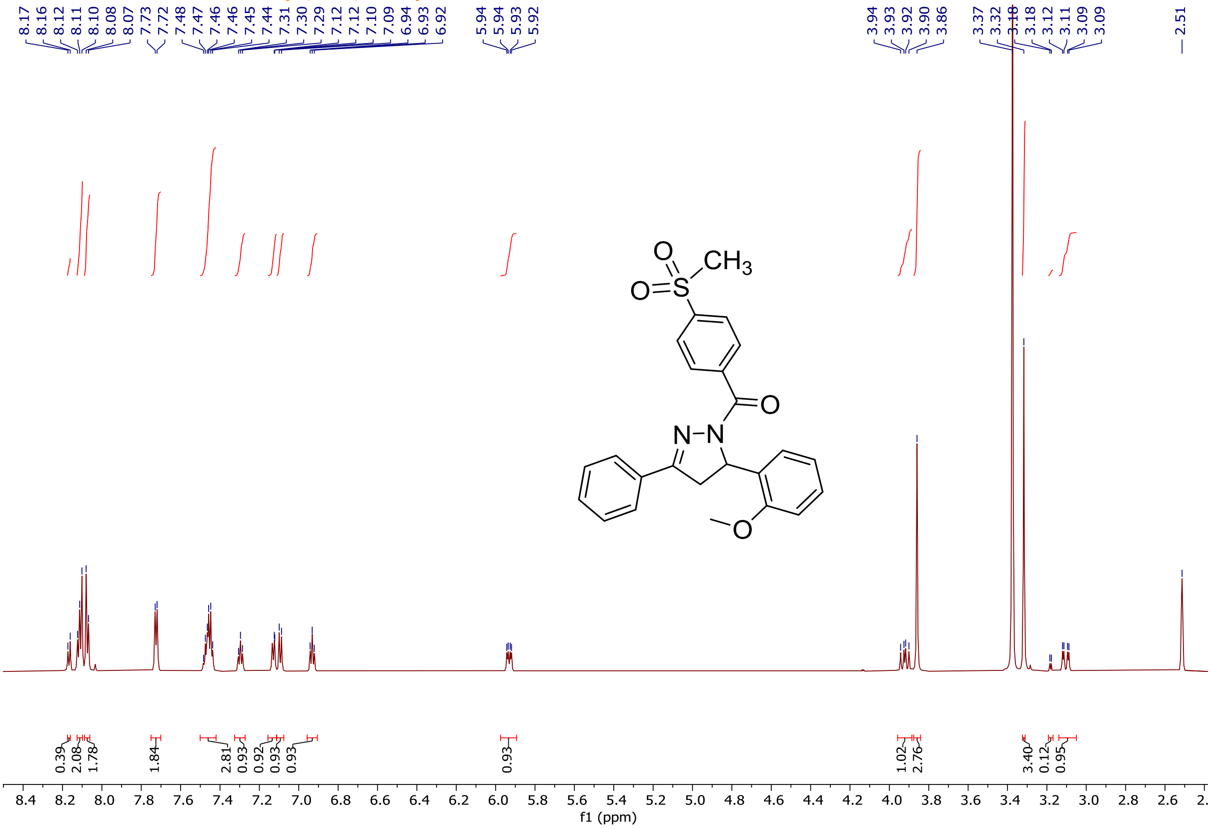

drmoenes-MS-24.11.fid — C13CPD DMSO {C:\Bruker\TOPSPIN} abari 52

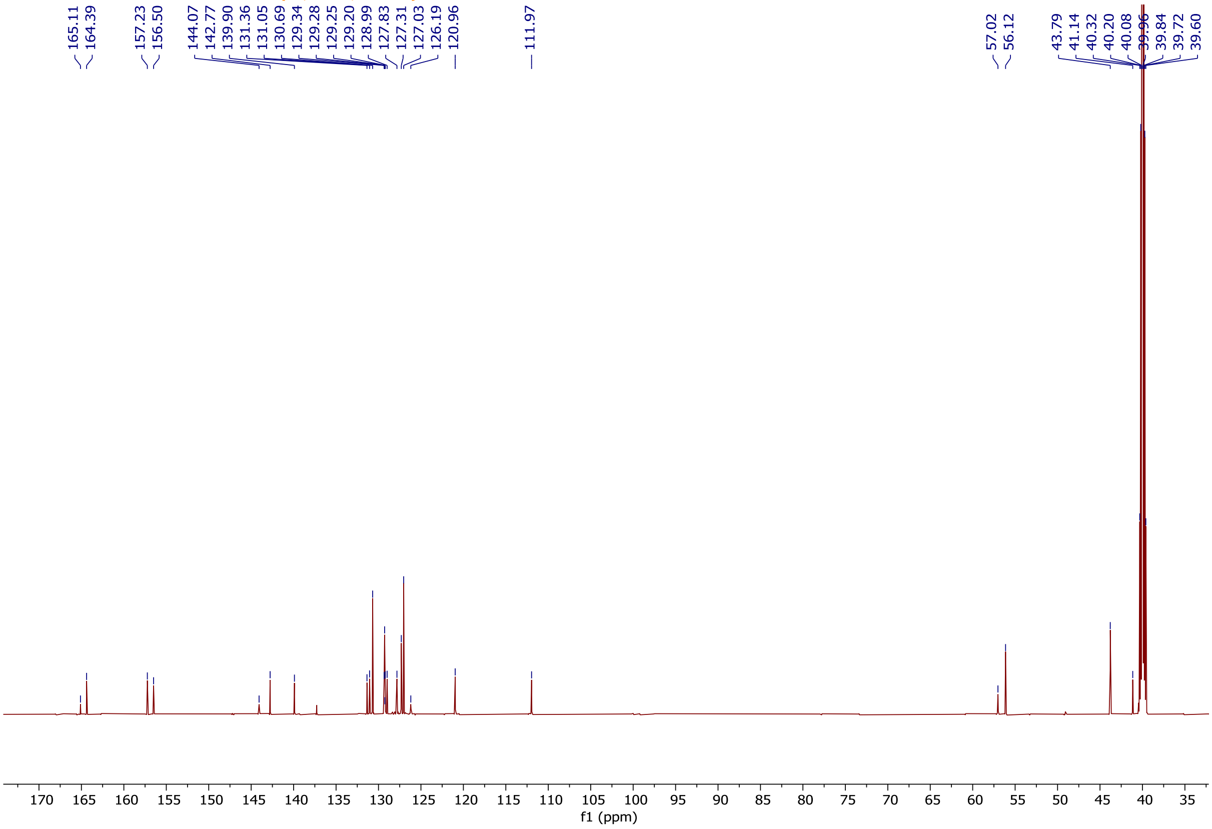

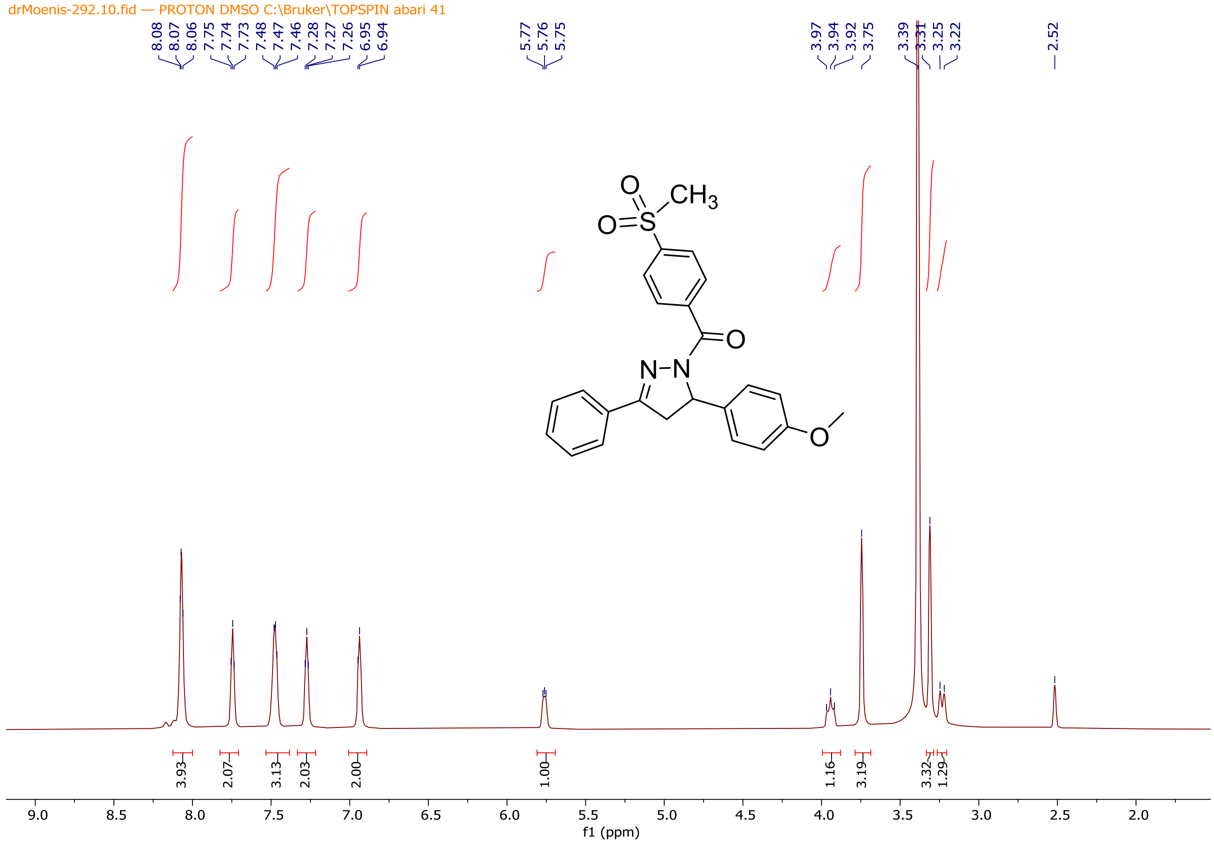
drMoenis-292.10.fid — PROTON DMSO C:\Bruker\TOPSPIN abari 41
8.08
8.07
8.06
7.75
7.74
7.73
7.48
7.47
7.46
7.28
7.27
7.26
6.95
6.94
5.77
5.76
5.75
3.97
3.94
3.92
3.75
3.39
3.31
3.25
3.22
2.52
O
CH3
O=S
O
N–N
O
3.93
2.07
3.13
2.03
2.00
1.00
1.16
3.19
3.32
1.29
9.0
8.5
8.0
7.5
7.0
6.5
6.0
5.5
5.0
4.5
4.0
3.5
3.0
2.5
2.0
f1 (ppm)

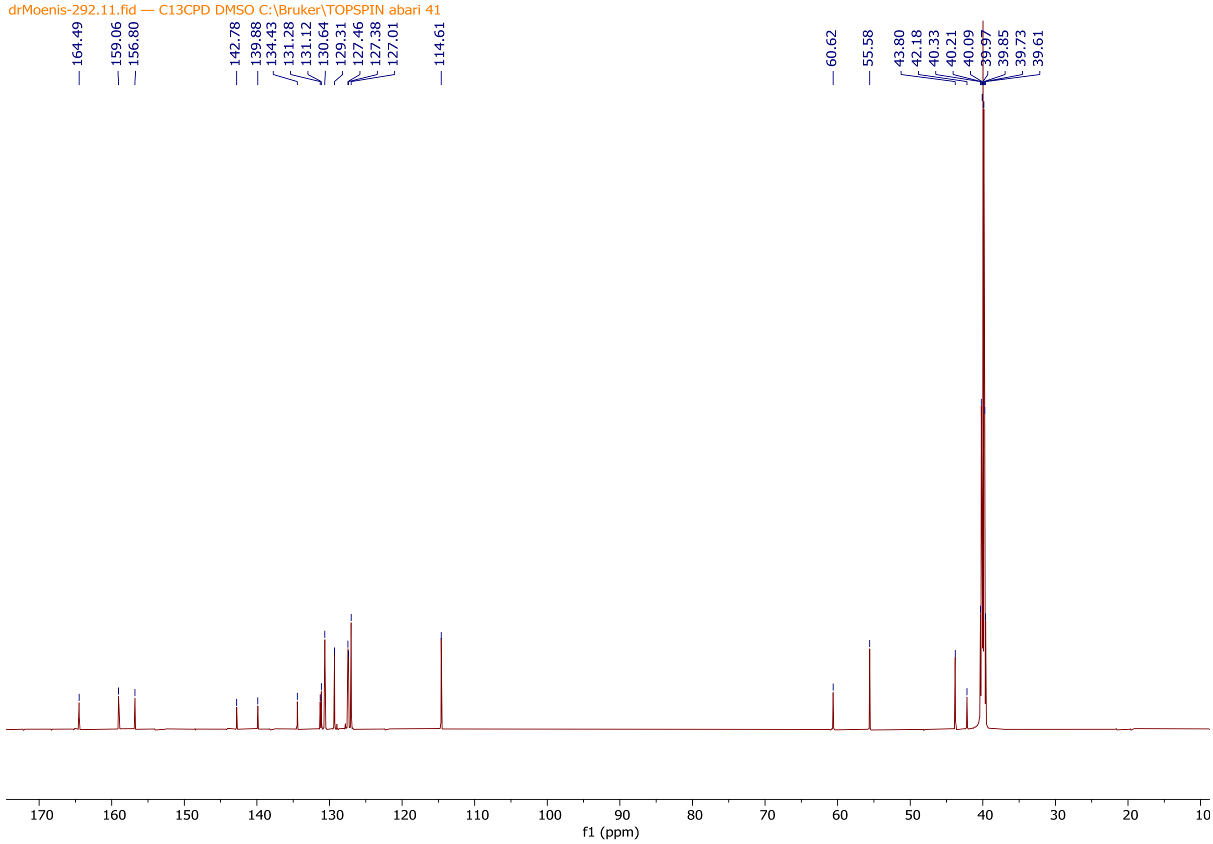
drMoenis-292.11.fid — C13CPD DMSO C:\Bruker\TOPSPIN abari 41
164.49
159.06
156.80
142.78
139.88
134.43
131.28
131.12
130.64
129.31
127.46
127.38
127.01
114.61
60.62
55.58
43.80
42.18
40.33
40.21
40.09
39.97
39.85
39.73
39.61
170
160
150
140
130
120
110
100
90
80
70
60
50
40
30
20
10
f1 (ppm)

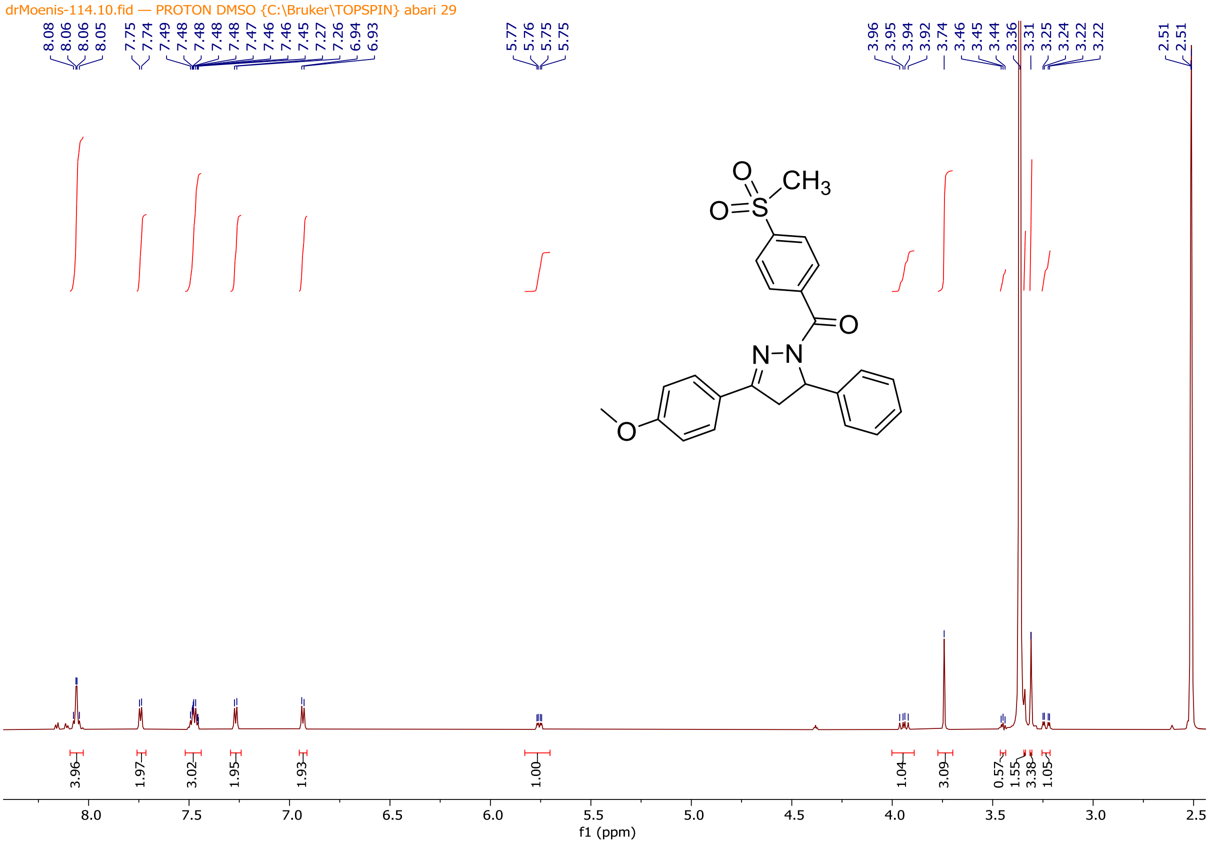
drMoenis-114.10.fid — PROTON DMSO {C:\Bruker\TOPSPIN} abari 29
8.08 8.06 8.06 8.05 7.75 7.74 7.49 7.48 7.48 7.48 7.47 7.46 7.46 7.45 7.27 7.26 6.94 6.93
5.77 5.76 5.75 5.75
3.96 3.95 3.94 3.92 3.74 3.46 3.45 3.44 3.36 3.31 3.25 3.24 3.22 3.22
2.51 2.51
CH3
O
O=S
O
N–N
O
3.96 1.97 3.02 1.95 1.93 1.00 1.04 3.09 0.57 1.55 3.38 1.05
8.0 7.5 7.0 6.5 6.0 5.5 5.0 4.5 4.0 3.5 3.0 2.5
f1 (ppm)

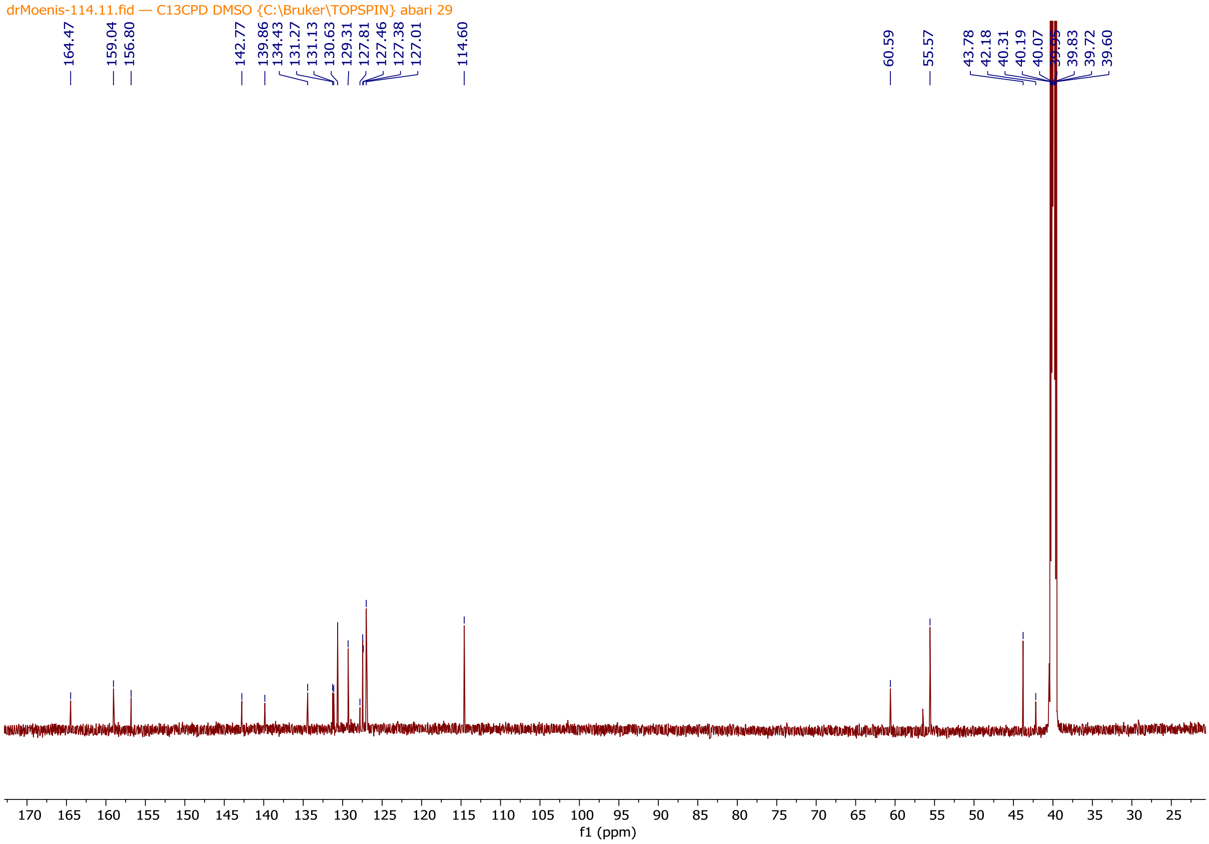
drMoenis-114.11.fid — C13CPD DMSO {C:\Bruker\TOPSPIN} abari 29
164.47 159.04 156.80
142.77 139.86 134.43 131.27 131.13 130.63 129.31 127.81 127.46 127.38 127.01
114.60
60.59 55.57
43.78 42.18 40.31 40.19 40.07 39.95 39.83 39.72 39.60
170 165 160 155 150 145 140 135 130 125 120 115 110 105 100 95 90 85 80 75 70 65 60 55 50 45 40 35 30 25
f1 (ppm)

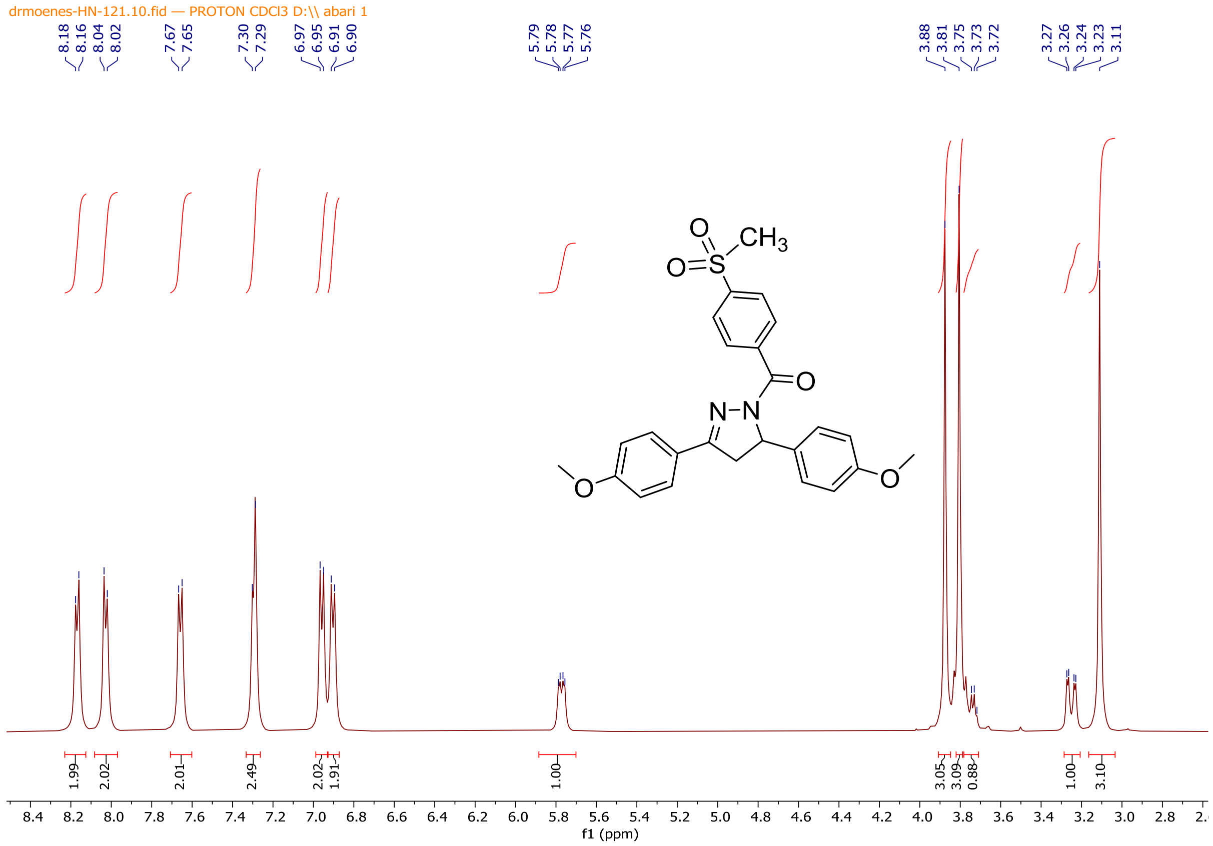
drmoenes-HN-121.10.fid — PROTON CDCl3 D:\\ abari 1
8.18 8.16 8.04 8.02 7.67 7.65 7.30 7.29 6.97 6.95 6.91 6.90
5.79 5.78 5.77 5.76
3.88 3.81 3.75 3.73 3.72
3.27 3.26 3.24 3.23 3.11
1.99 2.02 2.01 2.49 2.02 1.91 1.00 3.05 3.09 0.88 1.00 3.10
O
CH3
O=S
O
N–N
O
O
f1 (ppm)

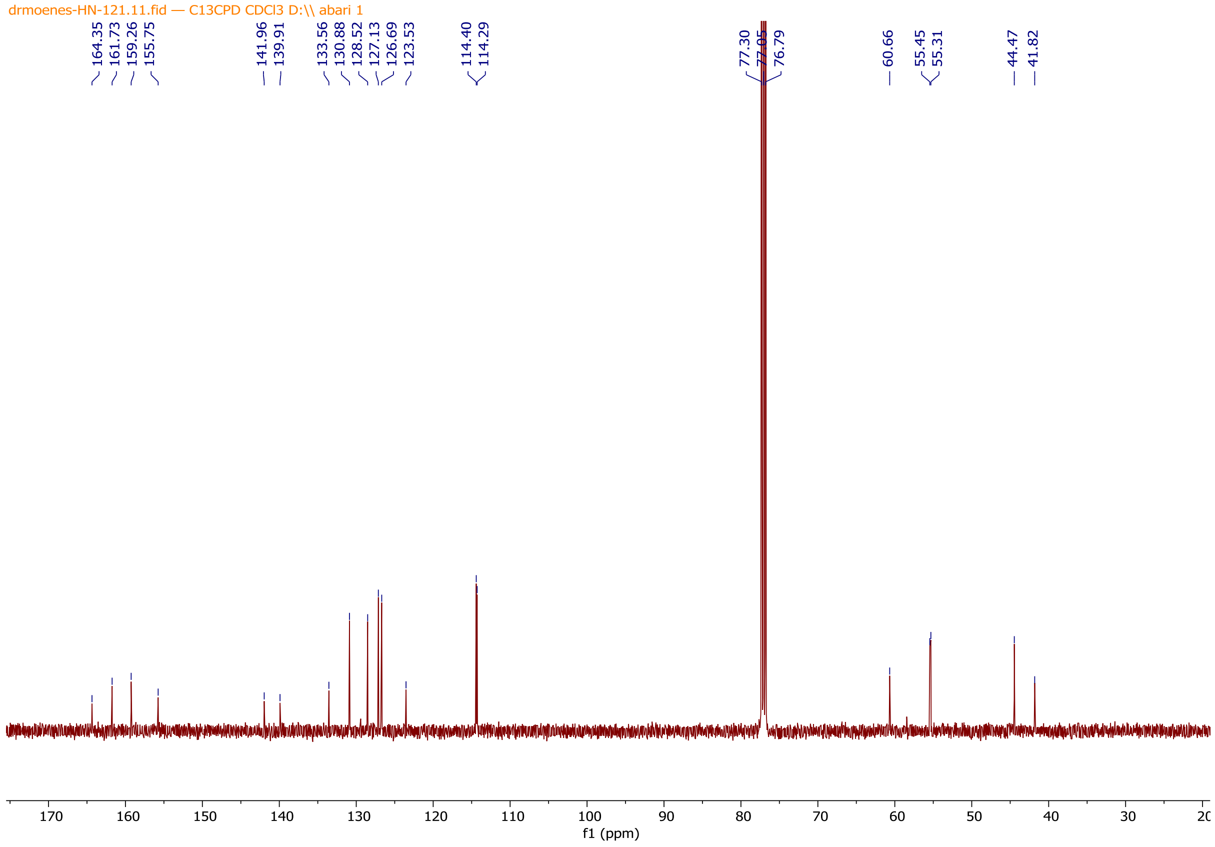
drmoenes-HN-121.11.fid — C13CPD CDCl3 D:\\ abari 1
164.35 161.73 159.26 155.75
141.96 139.91
133.56 130.88 128.52 127.13 126.69 123.53
114.40 114.29
77.30 77.05 76.79
60.66
55.45 55.31
44.47 41.82
f1 (ppm)

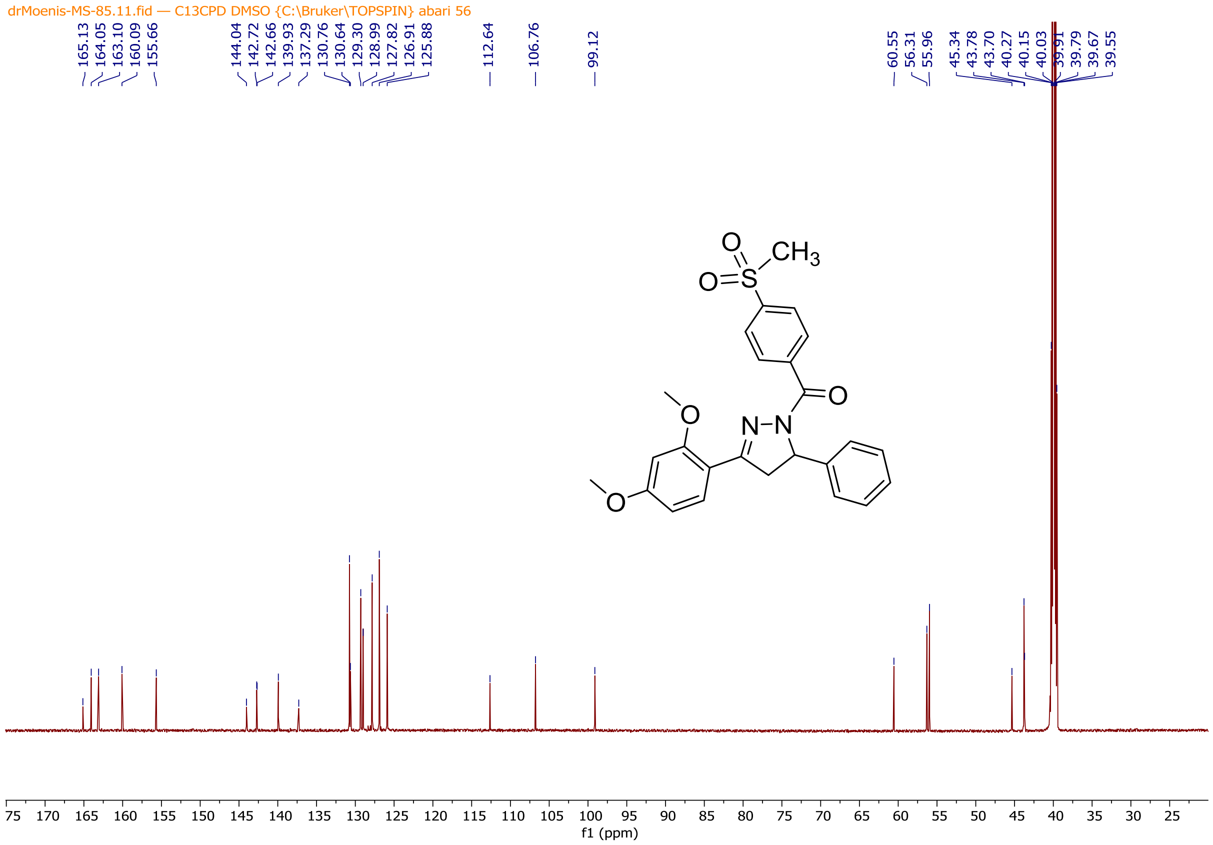
drMoenis-MS-85.11.fid — C13CPD DMSO {C:\Bruker\TOPSPIN} abari 56
165.13
164.05
163.10
160.09
155.66
144.04
142.72
142.66
139.93
137.29
130.76
130.64
129.30
128.99
127.82
126.91
125.88
112.64
106.76
99.12
60.55
56.31
55.96
45.34
43.78
43.70
40.27
40.15
40.03
39.91
39.79
39.67
39.55
O
CH3
O=S
O
N–N
O
O
75 170 165 160 155 150 145 140 135 130 125 120 115 110 105 100 95 90 85 80 75 70 65 60 55 50 45 40 35 30 25
f1 (ppm)

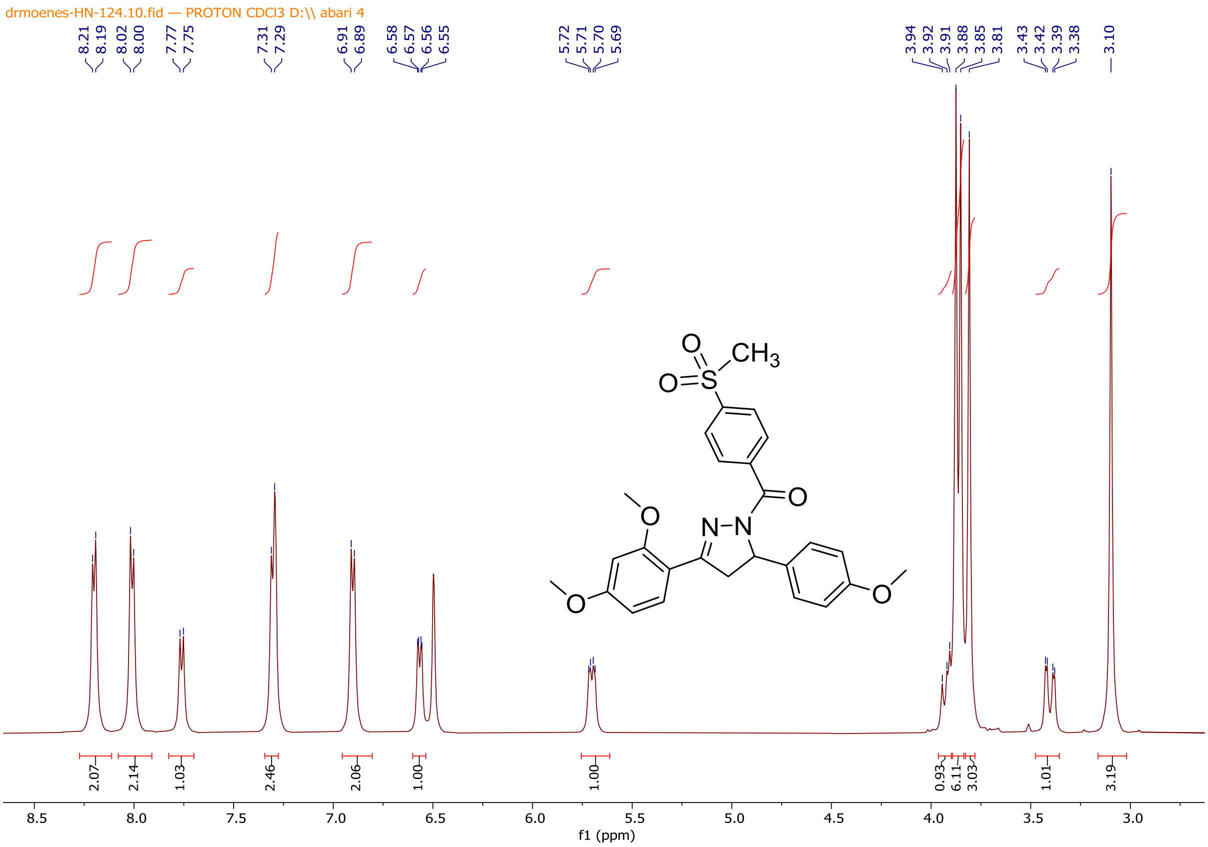
drmoenes-HN-124.10.fid — PROTON CDCl3 D:\\ abari 4
8.21 8.19 8.02 8.00 7.77 7.75 7.31 7.29 6.91 6.89 6.58 6.57 6.56 6.55 5.72 5.71 5.70 5.69 3.94 3.92 3.91 3.88 3.85 3.81 3.43 3.42 3.39 3.38 3.10
2.07 2.14 1.03 2.46 2.06 1.00 1.00 0.93 6.11 3.03 1.01 3.19
O
CH3
O=S
O
N–N
O
O
O
8.5 8.0 7.5 7.0 6.5 6.0 5.5 5.0 4.5 4.0 3.5 3.0
f1 (ppm)

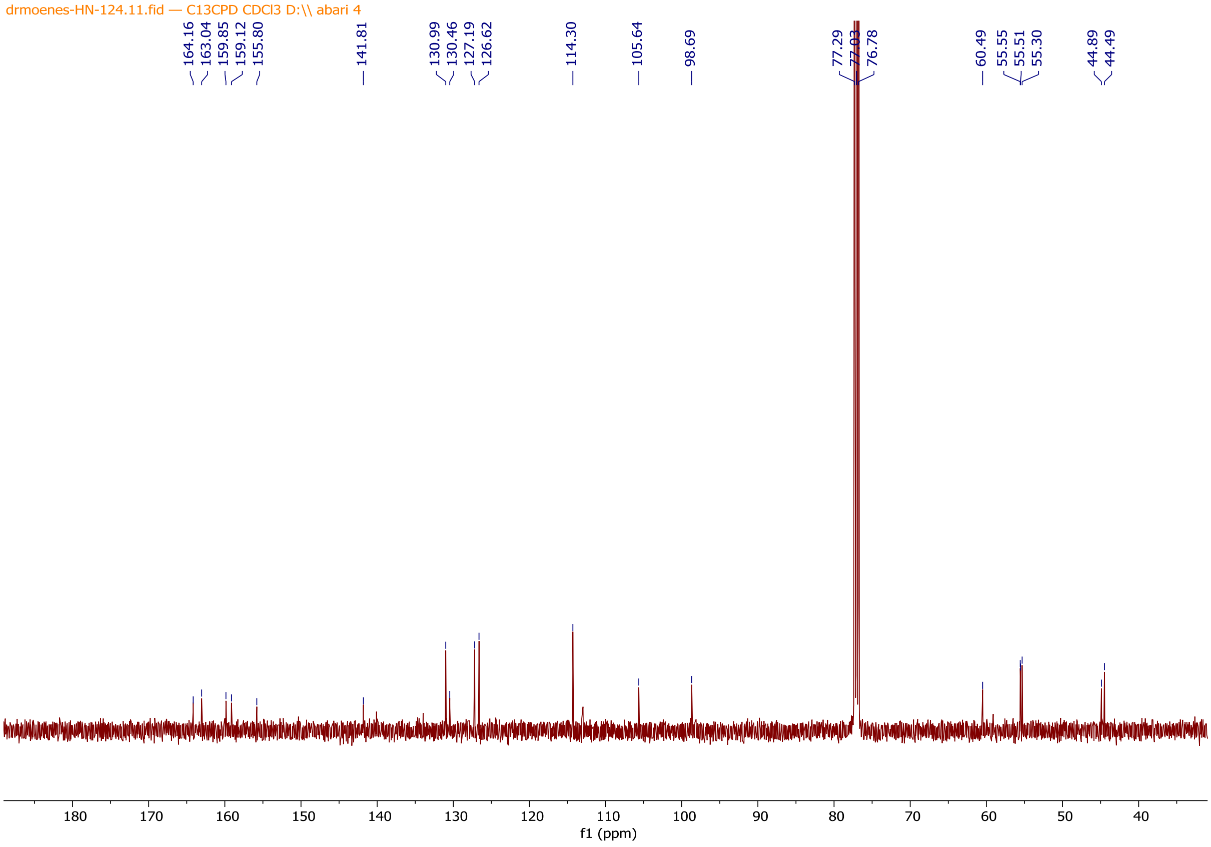
drmoenes-HN-124.11.fid — C13CPD CDCl3 D:\\ abari 4
164.16 163.04 159.85 159.12 155.80 141.81 130.99 130.46 127.19 126.62 114.30 105.64 98.69 77.29 77.03 76.78 60.49 55.55 55.51 55.30 44.89 44.49
180 170 160 150 140 130 120 110 100 90 80 70 60 50 40
f1 (ppm)

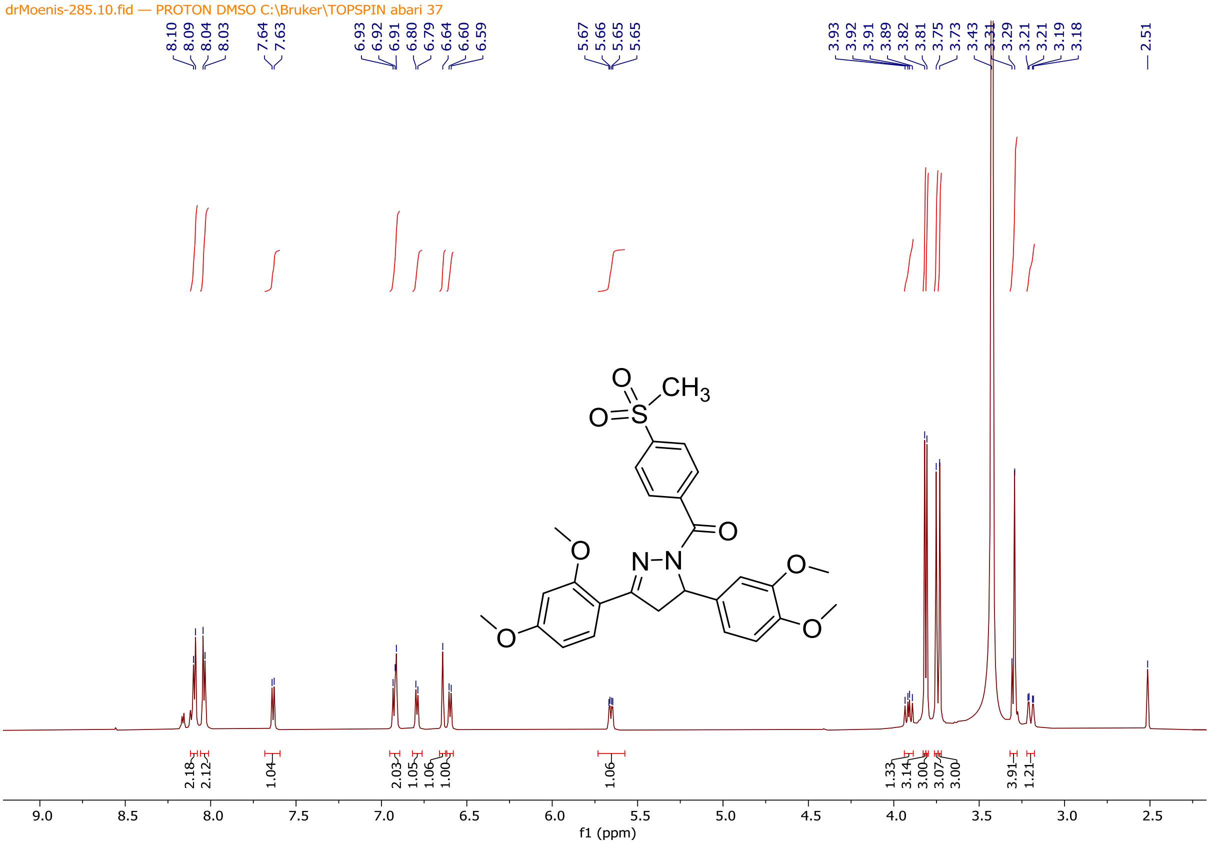
drMoenis-285.10.fid — PROTON DMSO C:\Bruker\TOPSPIN abari 37
8.10 8.09 8.04 8.03 7.64 7.63 6.93 6.92 6.91 6.80 6.79 6.64 6.60 6.59 5.67 5.66 5.65 5.65 3.93 3.92 3.91 3.89 3.82 3.81 3.75 3.73 3.43 3.31 3.29 3.21 3.21 3.19 3.18 2.51
O
CH3
O=S
O
N–N
O
O
O
O
2.18 2.12 1.04 2.03 1.05 1.06 1.00 1.06 1.33 3.14 3.00 3.07 3.00 3.91 1.21
9.0 8.5 8.0 7.5 7.0 6.5 6.0 5.5 5.0 4.5 4.0 3.5 3.0 2.5
f1 (ppm)

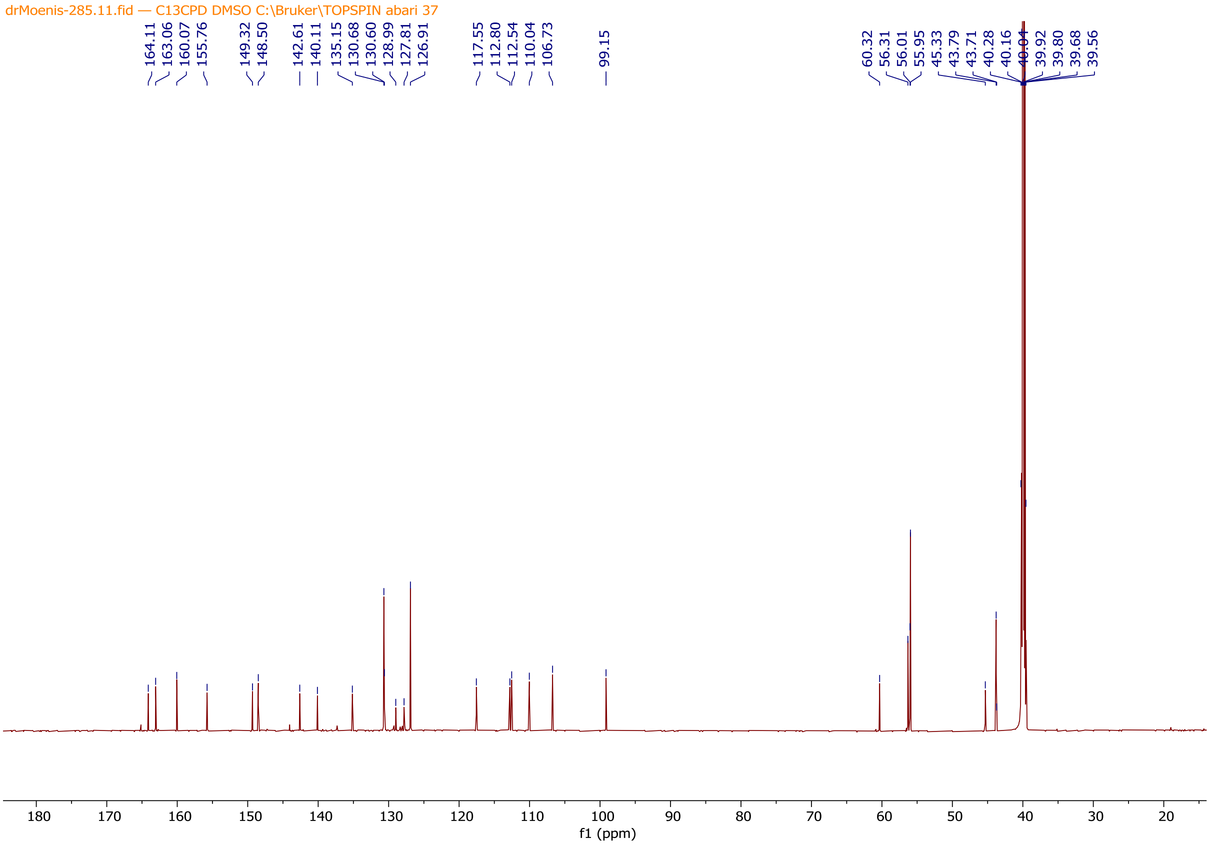
drMoenis-285.11.fid — C13CPD DMSO C:\Bruker\TOPSPIN abari 37
164.11 163.06 160.07 155.76 149.32 148.50 142.61 140.11 135.15 130.68 130.60 128.99 127.81 126.91 117.55 112.80 112.54 110.04 106.73 99.15 60.32 56.31 56.01 55.95 45.33 43.79 43.71 40.28 40.16 40.04 39.92 39.80 39.68 39.56
180 170 160 150 140 130 120 110 100 90 80 70 60 50 40 30 20
f1 (ppm)

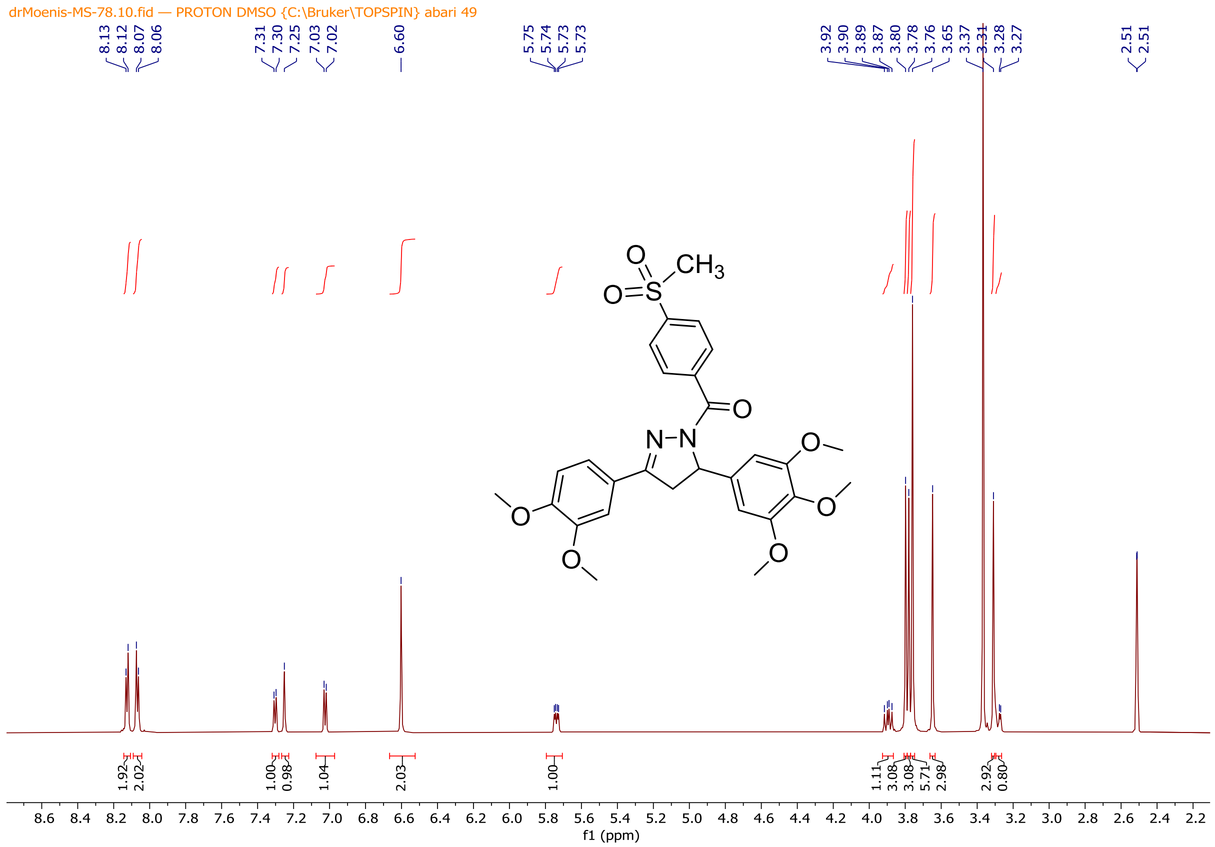
drMoenis-MS-78.10.fid — PROTON DMSO {C:\Bruker\TOPSPIN} abari 49
8.13 8.12 8.07 8.06 7.31 7.30 7.25 7.03 7.02 6.60 5.75 5.74 5.73 5.73 3.92 3.90 3.89 3.87 3.80 3.78 3.76 3.65 3.37 3.31 3.28 3.27 2.51 2.51
O
CH3
O=S
O
N–N
O
O
O
O
O
1.92 2.02 1.00 0.98 1.04 2.03 1.00 1.11 3.08 3.08 5.71 2.98 2.92 0.80
8.6 8.4 8.2 8.0 7.8 7.6 7.4 7.2 7.0 6.8 6.6 6.4 6.2 6.0 5.8 5.6 5.4 5.2 5.0 4.8 4.6 4.4 4.2 4.0 3.8 3.6 3.4 3.2 3.0 2.8 2.6 2.4 2.2
f1 (ppm)

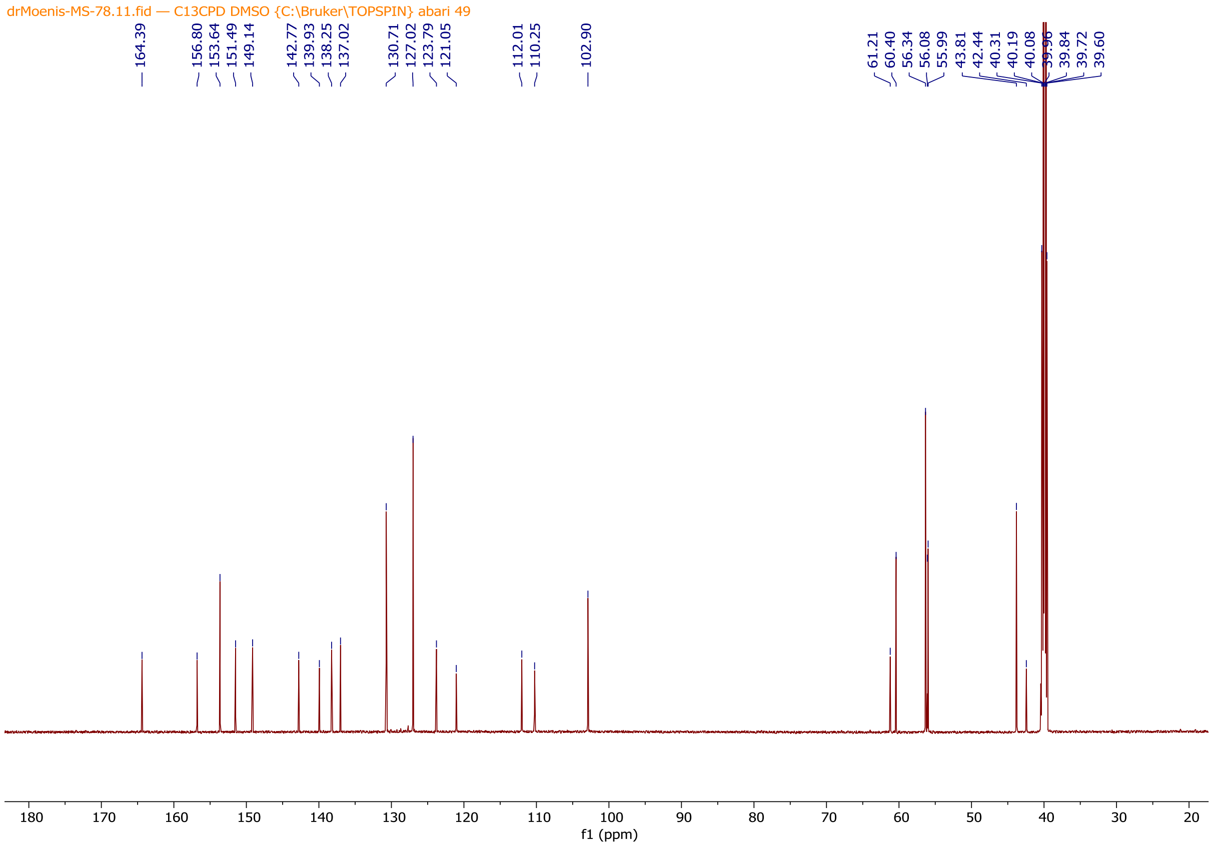
drMoenis-MS-78.11.fid — C13CPD DMSO {C:\Bruker\TOPSPIN} abari 49
164.39 156.80 153.64 151.49 149.14 142.77 139.93 138.25 137.02 130.71 127.02 123.79 121.05 112.01 110.25 102.90 61.21 60.40 56.34 56.08 55.99 43.81 42.44 40.31 40.19 40.08 39.96 39.84 39.72 39.60
180 170 160 150 140 130 120 110 100 90 80 70 60 50 40 30 20
f1 (ppm)
